# Retinal Microvascular Changes Associated with Concurrent Morphologic/Hematologic Response to Induction Therapy in Acute Leukemia: An Exploratory Prospective Wide-Field Swept-Source OCT Angiography Study

**DOI:** 10.3390/diagnostics16182940

**Published:** 2026-09-11

**Authors:** Jiawei Liu, Li Zhang, Jiamei Yang, Longqian Liu, Meixia Zhang

**Affiliations:** Department of Ophthalmology, West China Hospital, Sichuan University, Chengdu 610041, China; 373202551@qq.com (J.L.); hx_zhang_li@163.com (L.Z.); 946356344@qq.com (J.Y.); liulq@scu.edu.cn (L.L.)

**Keywords:** acute leukemia, optical coherence tomography angiography, swept-source OCT, deep capillary plexus, vessel density, candidate imaging measure, microcirculation, treatment response, choroid

## Abstract

**Background/Objectives:** Wide-field swept-source optical coherence tomography angiography (WF-SS-OCTA) enables non-invasive, layer-specific retinal microvascular assessment. We explored treatment-associated microvascular change in acute leukemia and its concurrent association with post-induction response. **Methods:** In this prospective single-center cohort (ChiCTR2300077558), 111 newly diagnosed adults (58 AML, 53 ALL) and 40 healthy controls underwent pre-induction 16 × 16 mm WF-SS-OCTA; 77 were re-imaged at first response assessment. Response (complete remission (CR)/CRi vs. partial/no response (PR/NR)) was adjudicated independently of OCTA. The focal exploratory outcome was deep capillary plexus vessel density change (ΔDCP-VD). This outcome was designated after completion of data collection, and all analyses are exploratory and hypothesis-generating. **Results:** DCP-VD showed an AML < ALL < control baseline gradient (42.49 ± 3.15%, 43.45 ± 2.89%, 44.27 ± 2.78%; ANOVA *p* = 0.014), and 10 of 20 baseline metrics survived false-discovery-rate correction. ΔDCP-VD was higher in CR/CRi than PR/NR (1.50 (1.00, 2.00) vs. 0.50 ± 0.83 percentage points; difference 0.97, 95% CI 0.57–1.37; *p* < 0.01) and correlated with hemoglobin change (r_s_ = +0.546, *p* < 0.01); in-sample discrimination gave an AUC of 0.799 (95% CI 0.674–0.907). **Conclusions:** ΔDCP-VD differed by concurrent response and tracked hemoglobin change; findings establish no causality, prediction, or clinical threshold and require independent prospective validation.

## 1. Introduction

Acute leukemia is a group of malignant clonal disorders of hematopoietic stem or progenitor cells in which uncontrolled blast proliferation displaces normal hematopoiesis. Most patients present with anemia, thrombocytopenia, and abnormal leukocyte counts at diagnosis; these abnormalities may contribute to microvascular dysfunction through several mechanisms, including tissue hypoxia, leukostasis, altered blood rheology, and endothelial activation [1,2]. Microcirculatory dysfunction is therefore increasingly recognized as a systemic feature of acute leukemia, yet it is not captured by routine clinical assessment. In addition, red-cell and platelet transfusions—near-universal during induction therapy—may themselves influence OCTA measurements through acute changes in hemoglobin concentration and blood rheology [1,2]. Although bone marrow examination remains the reference standard for treatment response assessment in acute leukemia, its invasive nature limits repeated evaluation. An imaging-based measure that noninvasively characterizes treatment-associated microvascular change could, if prospectively validated, provide complementary research information where frequent marrow-based assessment is impractical; its potential clinical utility, if any, would lie in noninvasive assessment of treatment-associated microvascular change alongside scheduled response evaluations, although whether such a measure could precede, reduce, or alter marrow-based evaluation has not been tested.

Because retinal vessels can be directly visualized in vivo, retinal imaging provides a practical approach for investigating microvascular alterations associated with systemic disease [3]. Ocular involvement in leukemia has been documented for decades—classically as leukemic retinopathy with retinal hemorrhages, Roth spots, and venous tortuosity [4,5]; prospective series have linked fundus signs to hematologic parameters such as platelet count and hematocrit [6,7,8]. However, ophthalmoscopy and fundus photography reveal primarily late, macroscopic events; they cannot resolve the layer-specific capillary perfusion changes that plausibly precede visible signs.

Optical coherence tomography angiography (OCTA) enables noninvasive, depth-resolved quantification of retinal and choroidal microvascular flow [9,10]. Swept-source instruments operating near 1050 nm penetrate to the choroid and acquire 100,000–400,000 A-scans per second, allowing wide-field (16 × 16 mm) single-scan coverage of the macula and optic disc [11,12]. Several quantitative OCTA parameters—vessel density, foveal avascular zone (FAZ) metrics, skeletonized network morphology, and choroidal vascular indices [13,14,15,16,17] have been validated for repeatability and applied across systemic diseases, in which deep capillary plexus (DCP) metrics have frequently been reported as sensitive indicators of microvascular stress [18,19].

In acute leukemia specifically, OCTA evidence remains preliminary: published studies enrolled 12–52 patients and were mostly cross-sectional or restricted to the macula [20,21,22,23]; an ultra-widefield OCTA study in remission-phase patients has recently been reported [24], but no study has compared AML with ALL, or combined 16 × 16 mm wide-field scanning with same-eye pre- and post-induction imaging anchored to response assessment or skeletonized morphology analysis [20,21,22,23]. These gaps leave unanswered whether OCTA-derived microvascular metrics can serve as candidate imaging measures of leukemia-associated microvascular state and treatment-associated change in acute leukemia.

We therefore conducted a prospective longitudinal cohort study using a 16 × 16 mm WF-SS-OCTA to determine (1) whether adults with newly diagnosed acute leukemia exhibit quantifiable retinal and choroidal microvascular alterations at diagnosis; (2) whether these alterations change after the first induction therapy; and (3) whether the magnitude of change is associated with the concurrent treatment response and hematologic change. The main exploratory imaging outcome was the change in DCP vessel density (ΔDCP-VD) between pre-treatment baseline (T0) and the first response-assessment time point (T1), compared between patients achieving remission (complete remission (CR) or CR with incomplete hematologic recovery (CRi)) and those not achieving remission (partial remission (PR) or no response (NR)). ΔDCP-VD was selected as the main exploratory outcome after completion of data collection because it was the only vessel-density metric showing an ordered AML < ALL < healthy-control gradient with a nominally significant baseline between-group difference (Section 4.2); no confirmatory imaging endpoint was predefined, and all analyses are exploratory and hypothesis-generating.

## 2. Materials and Methods

### 2.1. Study Design and Participants

This single-center, prospective, longitudinal observational cohort study was conducted at West China Hospital, Sichuan University, between November 2023 and December 2025, and is reported following the STROBE guidelines [25]. Consecutive adults (≥18 years) with newly diagnosed, induction-naïve AML or ALL, confirmed by bone marrow morphology, flow cytometry, and cytogenetic/molecular studies per the World Health Organization 5th edition and the International Consensus Classification [26,27], were eligible. Emergency pre-T0 cytoreduction (hydroxyurea or leukapheresis), corticosteroid prephase in ALL, and transfusion before T0 were permitted when clinically required; these exposures were recorded, and T0 imaging therefore reflects the pre-induction disease state rather than an entirely untreated state. Key exclusion criteria were ocular diseases affecting OCTA quality or microvascular assessment (diabetic or hypertensive retinopathy, glaucoma, macular disease), secondary or relapsed leukemia, prior chemotherapy or hematopoietic stem cell transplantation, comorbid hypertension or diabetes, high myopia (axial length > 26.00 mm), and inadequate image quality. Forty healthy adults, frequency-matched by 10-year age band and sex, with normal blood counts and no ocular or major systemic disease, were recruited from the community and hospital staff volunteers as controls and underwent a single baseline examination. Healthy controls did not undergo bone marrow examination. Frequency matching was chosen to maintain feasibility while preserving comparable demographic distributions between groups. Sample-size considerations at the design stage targeted the baseline cross-sectional comparisons: assuming a between-group difference of 5 percentage points in DCP-VD with a standard deviation of 6—chosen on the basis of clinical judgment and published OCTA measurement variability—23 participants per group would yield approximately 80% power at a two-sided alpha of 0.05; with allowance for an approximately 70% remission proportion and 20–30% attrition, enrollment of approximately 110 patients was planned. A design-context calculation for the longitudinal response-group comparison (a 3-percentage-point between-group difference in ΔDCP-VD with a standard deviation of 3; 16 patients per group) is provided only to inform future studies. Because the focal imaging outcome was designated after completion of data collection, these calculations are not a confirmatory sample-size justification for the response-group comparison; no observed (post hoc) power is reported, and uncertainty is expressed with 95% confidence intervals.

### 2.2. Ethics, Consent, and Registration

The study was conducted in accordance with the Declaration of Helsinki and was approved by the Biomedical Ethics Committee of West China Hospital, Sichuan University (approval No. 2023-(1753); date of approval: 17 October 2023). Written informed consent was obtained from all participants or their legal representatives, including consent for the publication of de-identified ocular images. No study-dedicated procedure was performed before ethics approval; the first participant was enrolled in November 2023; the temporal order of the first enrollment relative to registration on 13 November 2023 is documented in the original screening and consent records. The study was registered at the Chinese Clinical Trial Registry (ChiCTR2300077558; date of registration: 13 November 2023; chictr.org.cn). The registration did not define an imaging primary outcome; the focal imaging outcome and the analysis plan were designated in a statistical analysis plan (SAP) finalized after completion of data collection (26 July 2026, before the formal analyses were run), and missing-data sensitivity analyses were added in a revision of that plan (August 2026). All such post-registration analytical decisions are reported transparently as exploratory. Hematologic indices and response-adjudication bone marrow examinations were performed as part of routine clinical care at West China Hospital and abstracted into study case report forms; the values analyzed at T0 and T1 were those closest to the corresponding imaging examinations.

### 2.3. Observation Time Points and Response Assessment

Baseline imaging (T0) was completed within 72 h before the first dose of any antileukemic therapy. Follow-up imaging (T1) was anchored to the first response evaluation after one induction cycle, 28–56 days after T0 (median, 42 days) and within 7 days of the response-assessment bone marrow examination (observed median interval 2 days, interquartile range (IQR) 1–4, range −3 to +7 days, the scan occurring before or after the marrow examination). Response was adjudicated by hematologists independent of the imaging team who did not have access to OCTA data, with difficult cases resolved by senior review, according to the European LeukemiaNet 2022 recommendations for AML [28] and contemporary guidance for ALL [29,30]; the subtype-specific definitions were mapped onto a common operational categorization based exclusively on hematologic and bone marrow findings, categorized as complete remission (CR; < 5% bone marrow blasts, no extramedullary disease, and hematologic recovery with absolute neutrophil count ≥1.0 × 10^9^/L and platelet count ≥100 × 10^9^/L), CR with incomplete hematologic recovery (CRi; morphologic CR without full count recovery), partial remission (PR; ≥50% reduction in marrow blasts to 5–25%), or no response (NR; failure to meet these criteria). Measurable residual disease (MRD) was not incorporated into the operational response categories, which therefore denote morphologic/hematologic response rather than MRD-defined response. For the main analysis, CR and CRi formed the remission group (n = 54) and PR/NR the nonremission group (n = 23); the four-category distribution is reported in Section 3.6. All patients received standard first-line induction therapy according to contemporary guidelines; detailed regimen composition (specific cytarabine-anthracycline combinations, corticosteroids, asparaginase, and tyrosine kinase inhibitors) was not captured in the analysis dataset, whereas first-course anthracycline cumulative dose and transfusion volumes were recorded for all patients (Table S7). Hematologic indices (hemoglobin (Hb), white blood cell count (WBC), platelet count (PLT), peripheral and bone marrow blasts) and treatment exposures (red blood cell (RBC) and platelet transfusion volumes, first-course anthracycline cumulative dose by body surface area) were recorded at both time points; transfusion volumes were cumulative totals over the induction course (T0 to T1).

### 2.4. Ocular Examination and Wf-Ss-Octa Acquisition

Participants underwent best-corrected visual acuity, tonometry, axial length measurement, and dilated fundus examination with photography. Fundus findings (retinal hemorrhage graded on an ordinal 0–3 scale, where 0 indicates none and 1–3 indicate increasing extent and severity, the patient-level grade being the maximum across eyes; Roth spots, cotton-wool spots, vascular abnormalities, disc edema, macular involvement, leukemic infiltration) were recorded per eye and summarized at the patient level. Imaging used a 400 kHz swept-source OCTA device (BM-400K BMizar; TowardPi Medical Technology, Beijing, China) with a fixed 16 × 16 mm protocol centered on the fovea and covering the optic disc, acquired before pupillary dilation by trained technicians. This field of view was selected to prioritize quantitative repeatability for longitudinal comparison rather than maximal peripheral coverage. The device generated en face angiograms of the superficial (SCP) and deep (DCP) capillary plexuses, radial peripapillary capillaries (RPC), choriocapillaris, and medium/large choroidal vessel layers. Automated segmentation used manufacturer-defined slab boundaries (SCP: internal limiting membrane to the inner plexiform/inner nuclear layer (IPL/INL) boundary; DCP: IPL/INL boundary to the outer plexiform layer); the inner retinal vessel density (IRVD) was a separate device output quantifying the full inner retinal slab (internal limiting membrane to the outer plexiform layer), including superficial large-vessel contributions, rather than an arithmetic combination of SCP-VD and DCP-VD, with the device’s projection-resolved artifact-removal algorithm and motion correction enabled throughout; optic-disc, large-vessel, and low-signal/invalid-area masks were applied uniformly; regions affected by retinal hemorrhage were not manually masked, and the potential signal attenuation from hemorrhage on deeper-slab metrics is examined in a dedicated sensitivity analysis (Section 3.6), and vessel density was computed as the perfused-pixel area divided by the valid analyzed area. An image quality (IQ) score ≥ 7 (device scale 1–10) was required for inclusion; this operational rule, chosen from device documentation and published wide-field OCTA repeatability work [31], was applied throughout acquisition and formally consolidated in the current standard operating procedure after completion of data collection (Section 4.5); substandard scans were repeated on site (no more than two repeat scans per eye per time point) or excluded and logged. Artifact-handling rules excluded or corrected scans with motion artifacts involving > 5% of the analyzed area, uncorrectable large-vessel projection involving > 10%, segmentation boundary shifts > 20 μm or involving the central 3 mm, or invalid areas > 10% (Supplementary Method S2). Of the 302 baseline eyes scanned, 283 (93.7%; 203 patient eyes, 80 control eyes) passed quality control and entered the analysis; only eligible eyes were used throughout. All images were independently graded by two readers, with discrepancies resolved by senior adjudication. Between-eye consistency was evaluated with intraclass correlation coefficients (ICCs) [32,33,34,35], and patient-level analyses used the mean of eligible eyes. The full acquisition standard operating procedure, reader training, and quality-control pipeline are provided in Supplementary Methods S1 and S2, and the post-processing and choroidal quantification pipelines in Supplementary Methods S3 and S4.

### 2.5. Image Quantification

Device-generated metrics included inner retinal vessel density (IRVD), SCP-VD, DCP-VD, and RPC-VD (%), and FAZ area (mm^2^) and perimeter (mm) for the SCP, DCP, and whole-retinal slabs, all computed over the full 16 × 16 mm field after masking. Choroidal density metrics (choriocapillaris vessel density (VDCC); medium/large choroidal vessel density (VDMLCV)) were obtained from device angiographic outputs; volumetric choroidal indices (three-dimensional choroidal vascularity index (3D-CVI); choroidal vessel and stromal volume per unit area (CVV/a, CSV/a)) were generated by the device’s built-in software from automated choroidal segmentation and quantification, a device implementation of choroidal binarization and vascularity-index methodology [15,16]; and subfoveal choroidal thickness (SFCT) and central macular thickness (CMT) were measured on structural OCT (Supplementary Method S4). In addition, DCP en face images were post-processed in Fiji/ImageJ (Fiji 2.18.0 with ImageJ core 1.54p) with a fixed pipeline [36,37] with a fixed pipeline (background subtraction, Otsu binarization, optic-disc masking, skeletonization) to derive vessel length density (VLD), junction density [14,38], and box-counting fractal dimension (FD) [39], quantifying network topology beyond density alone as an exploratory morphology analysis (Supplementary Method S3). All images were analyzed at the nominal device scale (1024 × 1024 pixels = 16 × 16 mm; 15.625 μm/pixel) without individual axial-length magnification correction; eyes with axial length > 26.00 mm were excluded to limit scale error. Device-exported vessel-density values were integer percentages and volumetric choroidal indices were exported as integer micrometers; averaging one or two eligible eyes therefore yielded patient-level values on integer or 0.5-unit increments. No manufacturer-published nominal precision (test–retest variability) is available for vessel-density export under this 16 × 16 mm protocol.

### 2.6. Outcomes and Analysis Rules

The main exploratory imaging outcome was ΔDCP-VD, defined as DCP-VD at T1 minus DCP-VD at T0, computed with same-eye pairing (only eyes eligible at both time points; the patient-level value was the mean of eligible eyes) and compared between the remission and nonremission groups without multiplicity adjustment. The target estimand was the between-group mean difference (with 95% CI) in patient-level ΔDCP-VD between these groups in the same-eye qualified analysis set. The estimand is restricted to the 77 patients who completed same-eye paired imaging and is not extrapolated to all 111 enrolled patients. The main exploratory outcome and analysis plan were designated in a statistical analysis plan finalized after completion of data collection; accordingly, all analyses in this study are reported as exploratory. Secondary outcomes were the 12 SAP-designated longitudinal change outcomes (ΔIRVD, ΔSCP-VD, ΔRPC-VD, ΔDCP-FAZ area, Δwhole-FAZ area, ΔVDCC, ΔVDMLCV, Δ3D-CVI, ΔCVV/a, ΔDCP-VLD, ΔDCP-FD, and ΔDCP junction density). Per the statistical analysis plan, FAZ perimeters, SCP-FAZ area, CSV/a, SFCT, and CMT were designated cross-sectional (baseline) outcomes; longitudinal changes in the FAZ perimeters, SCP-FAZ area, CSV/a, and SFCT were additionally analyzed as a separate exploratory family (Section 3.3), whereas CMT was analyzed at baseline only; DCP morphologic metrics and all cross-sectional comparisons were likewise exploratory. Secondary and exploratory analyses were controlled for multiplicity using the Benjamini–Hochberg false discovery rate (FDR) [40], with families defined as the 20 baseline between-group comparisons, the 12 longitudinal secondary outcomes, and the five correlations between hematologic changes and ΔDCP-VD; raw *p* values and q values are reported for each family. As a sensitivity analysis addressing post hoc outcome selection, the focal outcome was additionally evaluated together with the 12 longitudinal secondary outcomes in a single 13-test Benjamini–Hochberg family (Section 3.3).

### 2.7. Statistical Analysis

Analyses followed the statistical analysis plan (finalized 26 July 2026, after completion of data collection; SPSS 26.0 (IBM, Armonk, NY, USA); Python 3.13 with NumPy, SciPy, and statsmodels; and R (pwr package) with PASS for sample-size verification; analysis code available upon reasonable request). Longitudinal analyses were complete-case analyses. Normality was assessed with the Shapiro–Wilk test; data are reported as mean ± standard deviation (SD) or median (interquartile range (IQR)). Three-group comparisons used one-way ANOVA with Bonferroni-adjusted post hoc tests or Kruskal–Wallis tests; two-group and paired comparisons used *t* tests or Mann–Whitney U tests and paired t or Wilcoxon tests, respectively, selected by Shapiro–Wilk normality assessment and reported in the corresponding table footnotes; categorical data used chi-square or Fisher exact tests. Correlations used Pearson coefficients for approximately normally distributed variables and Spearman coefficients for ordinal or non-normally distributed variables (Shapiro–Wilk test). Multivariable ordinary least squares regression estimated adjusted associations with ΔDCP-VD (variance inflation factors < 2 required), and model complexity was limited according to sample size considerations. A post hoc question concerned concurrent group separation: an exploratory receiver operating characteristic (ROC) analysis was performed to evaluate the ability of ΔDCP-VD to discriminate remission (CR/CRi) from nonremission (PR/NR), reporting the area under the curve (AUC) with bootstrap 95% confidence intervals (CIs; 5000 non-stratified resamples, percentile method; this interval quantifies sampling variability of the apparent AUC only and does not correct for selection optimism); because imaging and response were assessed concurrently at T1, this analysis reflects association rather than prediction and is explicitly framed as exploratory discrimination performance, not diagnostic accuracy. Sensitivity analyses comprised (1) multivariable adjustment (age, sex, baseline Hb, and image quality score) for the AML-versus-ALL baseline difference; (2) analysis of covariance (ANCOVA) modeling T1 DCP-VD with response group as the exposure and T0 DCP-VD, ΔHb, age, and sex as covariates; (3) eye-level models with patient-level clustering (cluster-robust standard errors) for the cross-sectional subtype difference and the longitudinal response-group difference, complemented by an eye-level longitudinal linear mixed-effects model (patient random intercept, maximum-likelihood estimation; time × response interaction); and (4) attrition-bias analysis comparing T1 completers versus patients lost to follow-up. Eye-level correlation analyses were verified with a patient-level cluster bootstrap (2000 resamples) to account for within-patient correlation of fellow eyes. The standardized effect size for the main outcome is reported as Hedges g, as designated in the statistical analysis plan. Descriptive statistics are reported as mean ± SD for cross-group comparability; inferential tests were selected by Shapiro–Wilk normality assessment (for example, baseline ALL DCP-VD departed from normality, Shapiro–Wilk *p* < 0.05, and between-group inference therefore used the Kruskal–Wallis test). Regression covariates (age, sex, baseline Hb) were specified in the post-data analysis plan before model fitting as demographic and severity-related potential confounders; post-treatment variables (ΔHb, transfusion volumes) were modeled as concurrent correlates, and their coefficients are interpreted as adjusted associations that cannot distinguish confounding from mediation or shared response-related change. Subgroup contrasts were tested directly between subgroups; no formal interaction terms were fitted, and within-subgroup significance was not used to infer between-subgroup differences. Missing data: longitudinal analyses were complete-case analyses by design. Post hoc sensitivity analyses for potentially informative attrition were added in the August 2026 revision of the analysis plan: (i) inverse probability weighting (IPW)—a logistic model for T1 completion in all 111 patients using baseline covariates (age, sex, subtype, Hb, WBC, PLT, marrow blasts, and baseline DCP-VD), stabilized weights truncated at the 1st/99th percentiles, with weight range, effective sample size, post-weighting standardized-mean-difference balance, and a robust-standard-error weighted between-group difference; (ii) multiple imputation by chained equations (MICE; m = 20) under a missing-at-random assumption, jointly imputing response status (logistic model) and ΔDCP-VD (linear model), combined with Rubin’s rules; and (iii) a δ-adjustment tipping-point analysis in which non-completers’ ΔDCP-VD was shifted by δ (0 to −15 percentage points in 0.25-point steps) relative to same-subtype completers, to identify the threshold δ* at which the main comparison would lose statistical significance (Section 3.6). There was no item-level missing data in the analysis dataset for the variables reported in the tables of Section 3; denominators for each analysis are the patient-level n shown in the corresponding tables (58/53/40 at baseline; 77/54/23 longitudinally). Image-level quality-control exclusions and participant-level loss to follow-up are distinct from item-level missing data and are reported in Section 3.1 (study flowchart). Two-sided *p* < 0.05 was considered significant. Throughout this report, *p* values are reported to three decimal places (values below 0.01 are denoted as *p* < 0.01), without asterisk annotation.

## 3. Results

### 3.1. Baseline Characteristics

**Figure 1:**
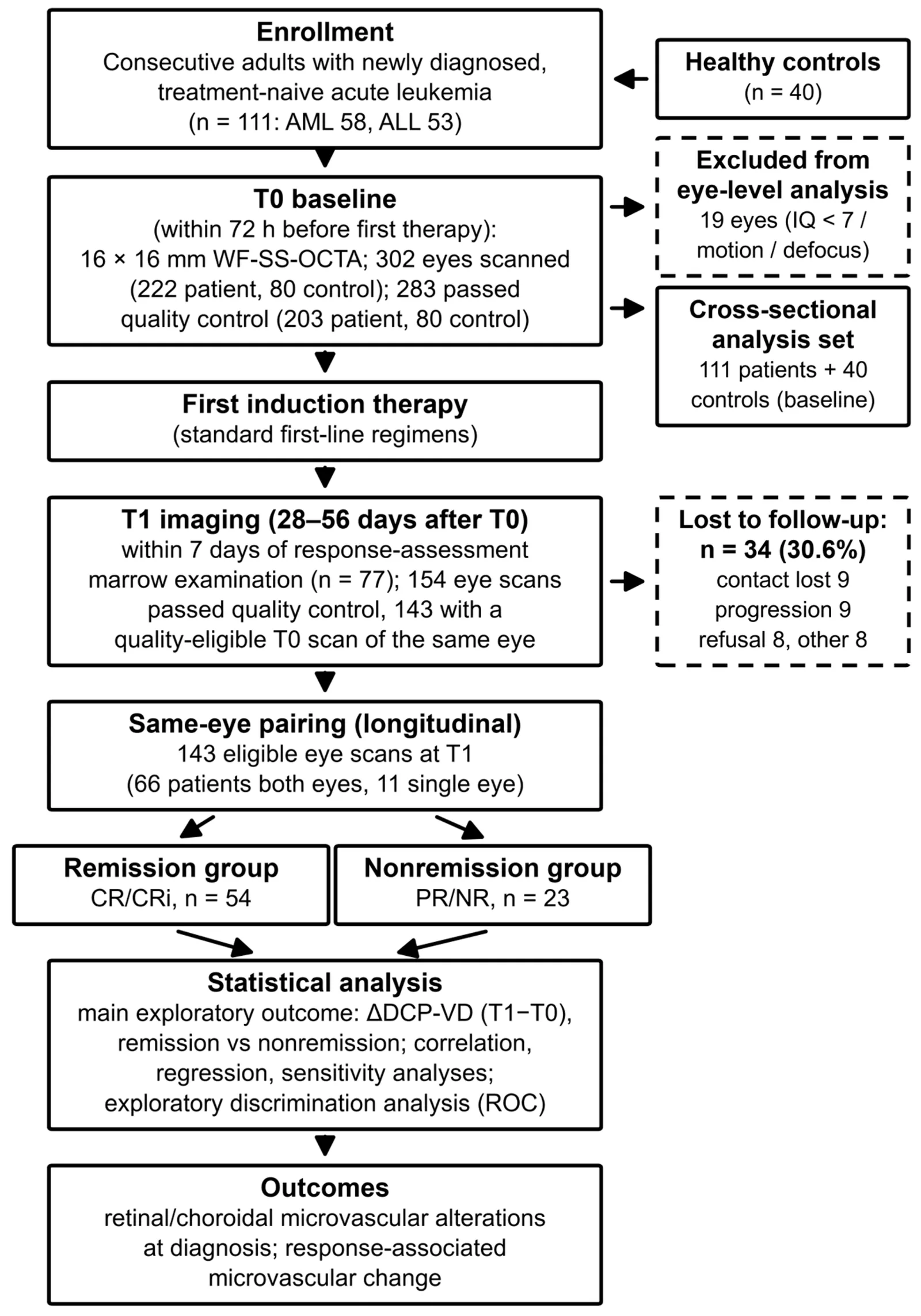
Study flowchart (STROBE format). Enrollment, T0 imaging with quality control (283/302 eyes analyzed), T1 follow-up with loss-to-follow-up reasons, same-eye pairing, response grouping (remission CR/CRi n = 54; nonremission PR/NR n = 23), and analysis sets. AML, acute myeloid leukemia; ALL, acute lymphoblastic leukemia; WF-SS-OCTA, wide-field swept-source optical coherence tomography angiography; IQ, image quality; CR, complete remission; CRi, complete remission with incomplete hematologic recovery; PR, partial remission; NR, no response.

A total of 111 adults with newly diagnosed acute leukemia (58 AML, 53 ALL) and 40 healthy controls (HCs) were enrolled (Figure 1). At T0, 302 eyes were scanned and 283 (93.7%; 203 patients, 80 controls) passed quality control and were analyzed. Seventy-seven patients (44 AML, 33 ALL) completed T1 imaging; 34 were lost to follow-up (30.6%; loss of contact, 9; disease progression/inability to undergo OCTA, 9; refusal, 8; other, 8). Fifty-four patients achieved composite remission (CR 25, CRi 29; composite remission proportion 70.1%) and 23 did not (PR 16, NR 7). The three groups did not differ in age (ANOVA *p* = 0.083), sex, visual acuity, intraocular pressure, or axial length (Table 1). Patients had lower Hb levels (86.19 ± 12.62 and 88.00 (79.00, 99.00) vs. 127.12 ± 6.70 g/L, *p* < 0.01) and PLT (42.00 (28.00, 64.00) and 49.19 ± 28.72 vs. 243.93 ± 61.88 × 10^9^/L, *p* < 0.01), and higher WBC and peripheral blast percentages than in HCs (both *p* < 0.01). Bone marrow examination was not performed in HCs, and hematologic profiles did not differ significantly between subtypes; treatment exposures during the T0–T1 interval (Table S7) were likewise similar between subtypes except for higher first-course anthracycline doses in the AML group (median 180 vs. 90 mg/m^2^, *p* < 0.01). Although no baseline variable differed significantly between T1 completers and non-completers (*p* range 0.129–1.000), small-to-moderate imbalances were present (absolute standardized mean differences up to 0.32 for subtype composition, 0.31 for Hb, and 0.27 for marrow blasts; Table S5), and 9 non-completions were related to progression or inability to undergo imaging; the losses were also clustered in the later enrollment-number segments, a pattern shown not to reflect administrative censoring (the last enrolled participant had passed the 28–56-day T1 window before database lock) and not otherwise fully explained. Missingness may therefore be informative, and complete-case longitudinal estimates cannot exclude residual attrition bias. Post hoc attrition sensitivity analyses (inverse probability weighting, multiple imputation, and δ-tipping scenarios) are reported in Section 3.6. Baseline characteristics of the two response groups are shown in Table 2: age, sex, AML/ALL subtype, baseline DCP-VD, Hb, transfusions, anthracycline dose, and follow-up interval were broadly comparable, with only platelet transfusion volume higher in the nonremission group (*p* = 0.040, SMD = −0.65; acknowledged as indication confounding and evaluated in multivariable models).

**Table 1:** Baseline demographic, ocular, and hematologic characteristics at T0.

Variable	AML (n = 58)	ALL (n = 53)	HC (n = 40)	*p* Value
Age, years	44.22 ± 11.15	39.53 ± 13.11	43.67 ± 10.47	0.083
Male, n (%)	28 (48.3)	33 (62.3)	18 (45.0)	0.188
BCVA, logMAR	0.05 (0.00, 0.05)	0.00 (−0.05, 0.05)	0.00 (0.00, 0.05)	0.622
IOP, mmHg	14.90 (13.53, 16.80)	15.10 (13.60, 16.70)	14.60 (13.25, 16.70)	0.828
Axial length, mm	23.77 (22.82, 24.46)	23.63 (22.80, 24.84)	23.57 (22.72, 24.42)	0.840
Hb, g/L	86.19 ± 12.62	88.00 (79.00, 99.00)	127.12 ± 6.70	<0.01
WBC, ×10^9^/L	9.00 (4.19, 17.33)	10.70 (5.36, 19.59)	5.84 ± 1.07	<0.01
PLT, ×10^9^/L	42.00 (28.00, 64.00)	49.19 ± 28.72	243.93 ± 61.88	<0.01
Peripheral blasts, %	15.35 (0.00, 21.70)	19.00 (0.00, 28.70)	0.00 ± 0.00	<0.01
Bone marrow blasts, %	56.83 ± 15.32	58.51 ± 14.68	Not assessed	0.557
T0-to-first-dose interval, h	36.90 ± 13.11	34.15 ± 13.43	-	0.278

**Table 2:** Clinical and treatment characteristics by response group (longitudinal cohort, n = 77).

Variable	Remission, CR/CRi (n = 54)	Nonremission, PR/NR (n = 23)	*p* Value
Age, years	39.74 ± 10.93	45.04 ± 13.67	0.075
AML, n (%)	31 (57.4)	13 (56.5)	1.000
Male, n (%)	30 (55.6)	12 (52.2)	0.982
T0 DCP-VD, %	42.75 ± 3.01	42.96 ± 3.06	0.785
T0 Hb, g/L	84.54 ± 11.72	90.48 ± 13.64	0.057
RBC transfusion, U	4.70 ± 2.57	4.22 ± 2.47	0.445
Platelet transfusion, doses	3.00 (2.00, 4.00)	4.30 ± 2.05	0.040
First-course anthracycline, mg/m^2^	120.00 (60.00, 135.00)	120.00 (60.00, 180.00)	0.718
T0-to-T1 interval, days	43.50 (34.00, 48.75)	40.39 ± 7.50	0.270

Data are mean ± SD or median (interquartile range). Three-group comparisons by one-way ANOVA or Kruskal–Wallis test. Treatment exposures during the T0–T1 interval are process variables and are reported separately by subtype in Table S7 and by response group in Table 2. Categorical variables were compared with chi-square or Fisher exact tests, as appropriate. Bone marrow blasts were compared between AML and ALL only (independent-samples *t* test); bone marrow examination was not performed in healthy controls. T1 completers (n = 77) and patients lost to follow-up (n = 34) showed no statistically significant baseline differences (age *p* = 0.399; Hb *p* = 0.129; WBC *p* = 0.502; PLT *p* = 0.980; bone marrow blasts *p* = 0.186), although absolute standardized mean differences reached 0.32 (Table S5). AML, acute myeloid leukemia; ALL, acute lymphoblastic leukemia; HC, healthy control; BCVA, best-corrected visual acuity; IOP, intraocular pressure; Hb, hemoglobin; WBC, white blood cell count; PLT, platelet count; *p* values are reported to three decimal places (values below 0.01 are denoted as *p* < 0.01); no asterisk annotation is used.

Independent-samples *t* test or Mann–Whitney U test for continuous variables and chi-square or Fisher exact test for categorical variables, as appropriate; *p* values are reported to three decimal places (values below 0.01 are denoted as *p* < 0.01); no asterisk annotation is used. RBC, red blood cell; CR, complete remission; CRi, CR with incomplete hematologic recovery; PR, partial remission; NR, no response.

### 3.2. Baseline Microvascular Alterations

**Figure 2:**
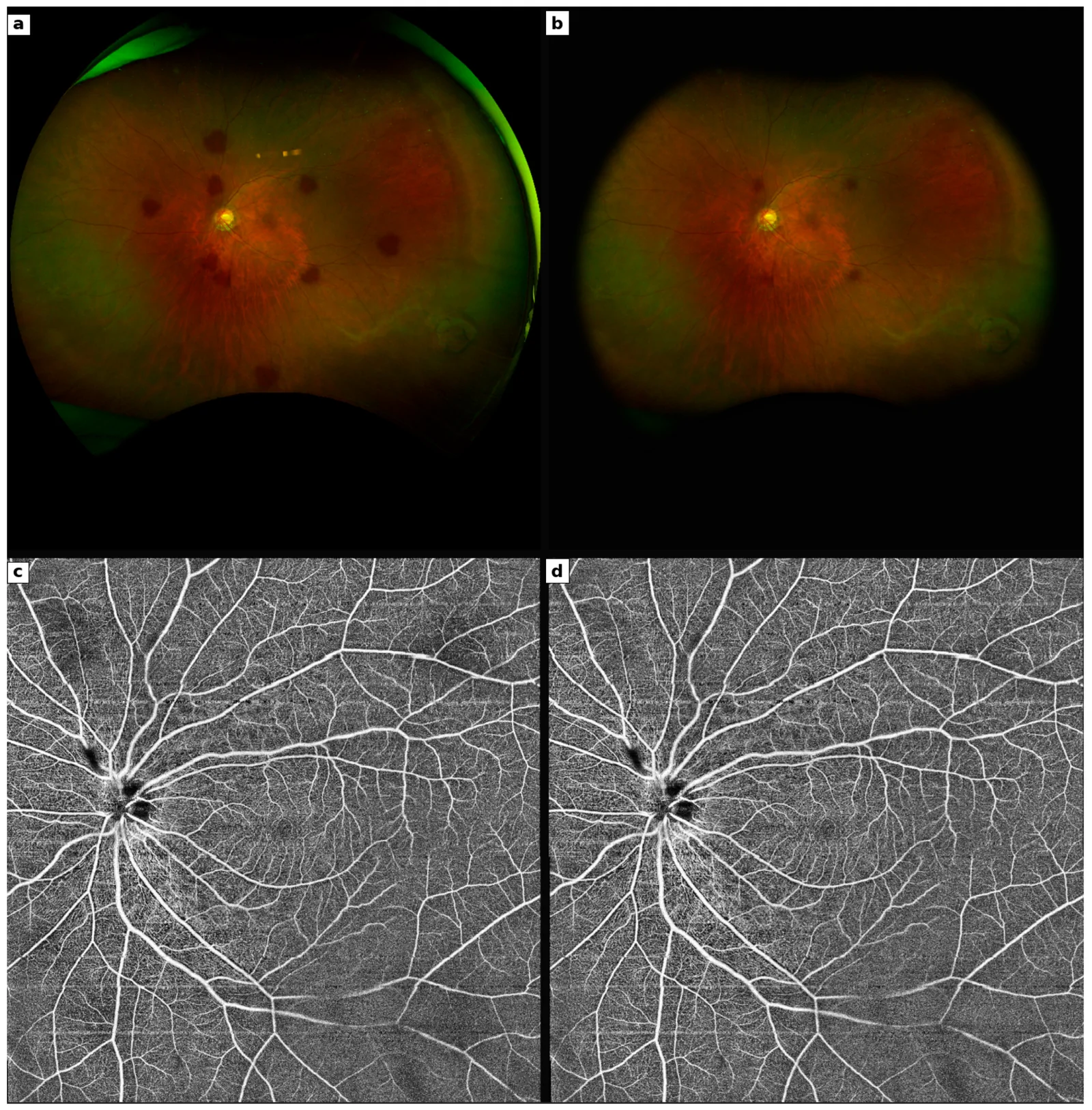
Representative multimodal imaging in a patient with newly diagnosed acute leukemia (same eye, identical fovea-centered protocol at both time points). (**a**,**b**) Ultra-widefield color fundus photographs at T0 (moderate retinal hemorrhages) and at T1 after achieving remission (visible hemorrhages were less extensive at T1 in this illustrative case). (**c**,**d**) Corresponding 16 × 16 mm WF-SS-OCTA retinal en face angiograms at T0 and T1. This single illustrative case is not intended to represent all patients; the eye laterality and time point of each panel were verified against the device export log, and only cropping and uniform brightness adjustment were applied. WF-SS-OCTA, wide-field swept-source optical coherence tomography angiography.

Conventional fundus signs at T0 were infrequent and mild (Table S1, Figure S1): retinal hemorrhage occurred in 19.8% of patients (22 patients/39 eyes; predominantly grade 1–2), while Roth spots, cotton-wool spots, disc edema, macular involvement, and leukemic infiltration were uncommon, with no AML-versus-ALL differences (all *p* > 0.05). Hemorrhage grade correlated negatively with baseline Hb (Spearman r_s_ = −0.316, *p* < 0.01) and with DCP-VD (patient level r_s_ = −0.280, *p* < 0.01). Representative multimodal imaging from a single illustrative patient, showing ultra-widefield color fundus photographs and 16 × 16 mm WF-SS-OCTA angiograms at both time points, is presented in Figure 2.

**Figure 3:**
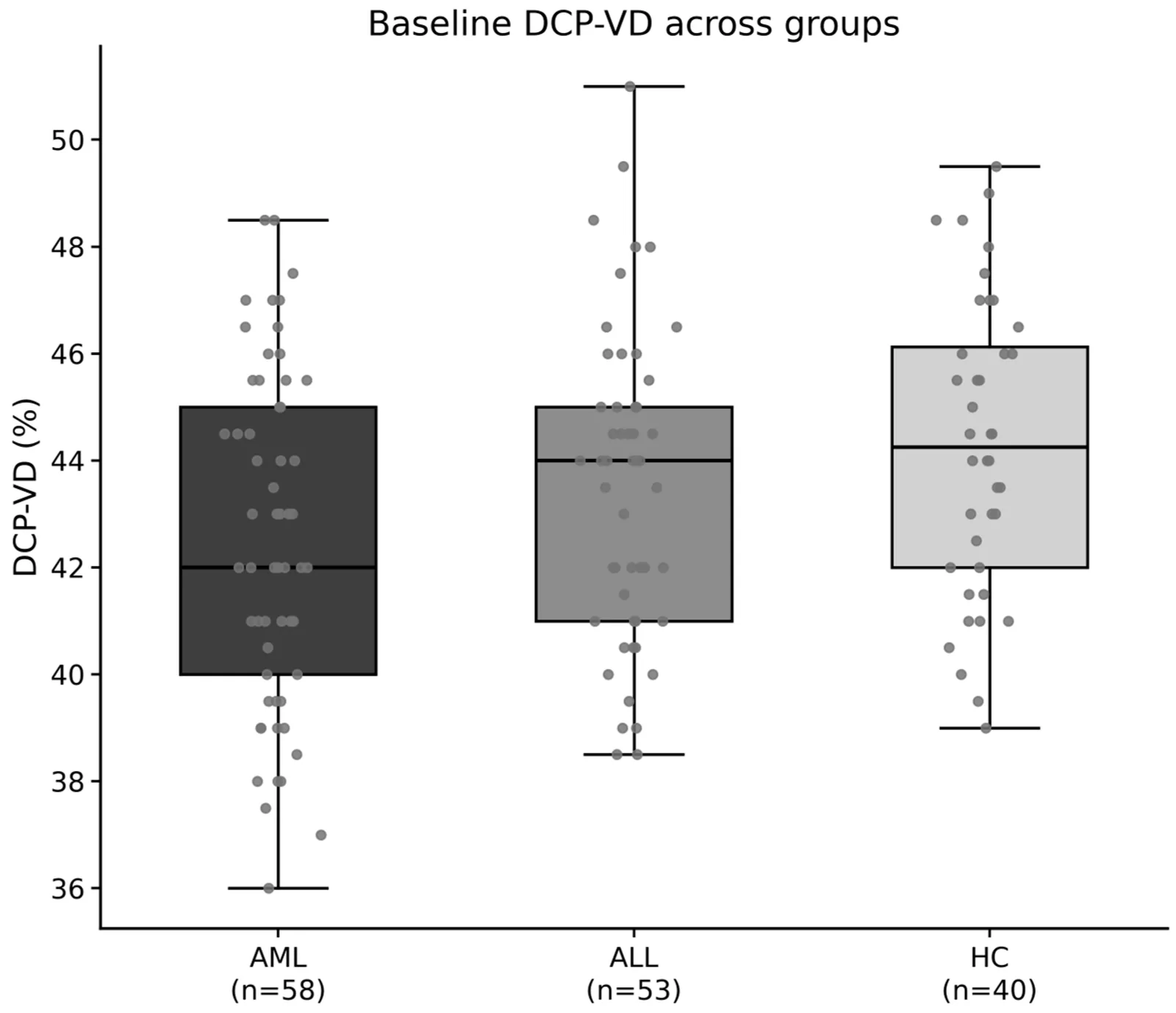
Baseline DCP vessel density across groups. Boxes show the median and interquartile range (IQR) with 1.5× IQR whiskers; dots are individual patient-level values; quoted values are mean ± SD. Patient-level DCP-VD in acute myeloid leukemia (AML; n = 58), acute lymphoblastic leukemia (ALL; n = 53), and healthy controls (HC; n = 40): 42.49 ± 3.15% vs. 43.45 ± 2.89% vs. 44.27 ± 2.78%; one-way ANOVA *p* = 0.014, η^2^ = 0.056; only the AML-versus-HC pairwise comparison was significant after Bonferroni adjustment (*p* = 0.014). DCP-VD, deep capillary plexus vessel density.

OCTA metrics at T0 showed broad between-group differences (Table 3). DCP-VD showed a numerical gradient—lowest in AML, intermediate in ALL, highest in HC (42.49 ± 3.15% vs. 43.45 ± 2.89% vs. 44.27 ± 2.78%; one-way ANOVA *p* = 0.014, η^2^ = 0.056)—with only the AML-versus-HC pairwise comparison significant after Bonferroni adjustment (*p* = 0.014; AML vs. ALL *p* = 0.294; ALL vs. HC *p* = 0.513; group distributions in Figure 3). Patient-level between-group differences were 0.96 percentage points (95% CI −0.18–2.10; ALL minus AML), 0.82 (−0.36–2.01; HC minus ALL), and 1.78 (0.56–3.01; HC minus AML). SCP-VD (*p* < 0.01), IRVD (*p* = 0.013), DCP-FAZ area (*p* < 0.01), VDCC (*p* < 0.01), 3D-CVI (*p* < 0.01), CVV/a (*p* = 0.013), SFCT (*p* = 0.019), DCP-VLD (*p* < 0.01), and DCP-FD (*p* < 0.01) also differed across groups—generally with lower values in patients, most consistently in AML, than in controls—and DCP junction density differed nominally (*p* = 0.044; ALL vs. HC *p* = 0.035), whereas RPC-VD, central macular thickness, and the remaining FAZ and choroidal metrics did not (Tables S2–S4). After adjustment for age, sex, and baseline Hb, neither the AML-versus-ALL difference (B = −0.782, 95% CI −1.918 to +0.354, *p* = 0.176) nor the HC-versus-ALL difference (B = −0.171, 95% CI −2.169 to +1.827, *p* = 0.866) was significant, and age itself was not associated with DCP-VD (*p* = 0.808); an extended model adding image quality (AML vs. ALL B = −0.801, *p* = 0.169) and an eye-level model with patient-level clustering (B = −0.699, 95% CI −1.852 to +0.455, *p* = 0.235) gave consistent estimates. Ten of the 20 baseline comparisons survived Benjamini–Hochberg correction within the baseline family (q < 0.05: SFCT, IRVD, SCP-VD, DCP-VD, DCP-FAZ area, VDCC, 3D-CVI, CVV/a, DCP-VLD, and DCP-FD; DCP junction density q = 0.080), so the baseline cross-sectional associations constitute multiplicity-controlled but exploratory evidence of retinal and choroidal microvascular involvement at diagnosis, whereas the AML-versus-ALL gradient is not subtype-specific after covariate adjustment.

Ten of the 20 baseline comparisons remained significant after Benjamini–Hochberg FDR correction within the 20-comparison baseline family (q < 0.05: SFCT, IRVD, SCP-VD, DCP-VD, DCP-FAZ area, VDCC, 3D-CVI, CVV/a, DCP-VLD, and DCP-FD); DCP junction density did not (q = 0.080). For DCP-VD, only the AML-versus-HC pairwise difference was significant after Bonferroni adjustment (*p* = 0.014); patient-level between-group differences (95% CI): ALL minus AML 0.96 (−0.18–2.10), HC minus ALL 0.82 (−0.36–2.01), HC minus AML 1.78 (0.56–3.01) percentage points; η^2^ = 0.056 (whole-FAZ area η^2^ 0.027; SCP-VD ε^2^ 0.105; DCP-FAZ area ε^2^ 0.110; VDCC ε^2^ 0.057; 3D-CVI ε^2^ 0.073; CVV/a ε^2^ 0.045; IRVD ε^2^ 0.045; SFCT ε^2^ 0.040; DCP-VLD ε^2^ 0.142; DCP-FD ε^2^ 0.135). DCP junction density did not differ significantly between AML and ALL (Bonferroni-adjusted *p* = 1.000). Full FAZ, choroidal, and DCP morphology results are provided in Tables S2–S4. VD, vessel density; FAZ, foveal avascular zone; VDCC, choriocapillaris vessel density; 3D-CVI, three-dimensional choroidal vascularity index; SFCT, subfoveal choroidal thickness; VLD, vessel length density; FD, fractal dimension; *p* values are reported to three decimal places (values below 0.01 are denoted as *p* < 0.01); no asterisk annotation is used.

**Table 3:** Baseline (T0) wide-field SS-OCTA alterations across groups (key metrics; patient level, quality-controlled eyes).

Variable	AML (n = 58)	ALL (n = 53)	HC (n = 40)	*p* Value
DCP-VD, %	42.49 ± 3.15	43.45 ± 2.89	44.27 ± 2.78	0.014
SCP-VD, %	40.75 (39.00, 45.50)	41.00 (39.50, 44.00)	43.75 (42.38, 47.00)	<0.01
IRVD, %	40.50 (37.00, 44.00)	40.00 (38.00, 44.00)	43.50 (38.50, 46.62)	0.013
RPC-VD, %	46.74 ± 5.69	47.00 (42.00, 50.00)	48.00 (44.88, 50.00)	0.315
Whole-FAZ area, mm^2^	0.34 ± 0.05	0.34 ± 0.03	0.33 ± 0.03	0.133
VDCC, %	46.75 (40.62, 51.38)	46.50 (44.00, 52.50)	50.14 ± 4.63	<0.01
3D-CVI, %	33.50 (32.00, 34.00)	32.00 (29.00, 37.00)	34.99 ± 2.91	<0.01
SFCT, μm	256.00 (224.25, 268.88)	263.84 ± 42.74	261.50 (238.38, 287.88)	0.019
DCP-VLD, mm/mm^2^	8.56 (6.91, 9.90)	8.38 ± 1.75	9.92 ± 1.29	<0.01
DCP-FD	1.54 (1.37, 1.68)	1.55 (1.42, 1.70)	1.69 ± 0.05	<0.01
DCP junction density, n/mm^2^	35.84 ± 9.17	35.92 (30.05, 40.30)	38.66 (32.30, 46.67)	0.044

### 3.3. Longitudinal Changes After Induction Therapy: Main Exploratory Outcome

**Figure 4:**
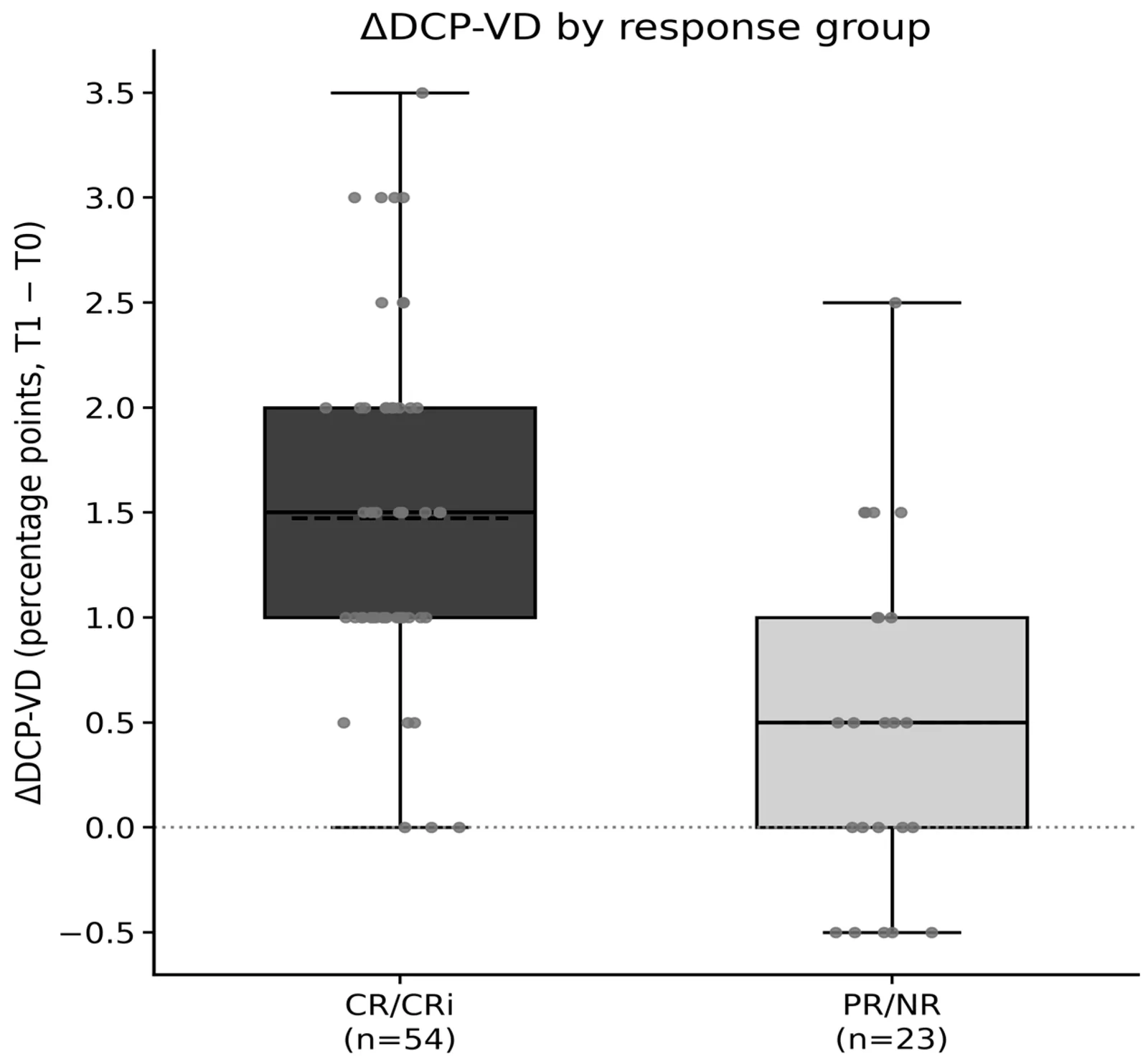
Change in DCP-VD after induction therapy by response group (same-eye pairing). Boxes show the interquartile range (IQR), the center line the median, and whiskers 1.5× IQR; each dot is a patient-level mean of quality-eligible same-eye pairs; the dashed line within each box marks the group mean, and the dotted line marks no change (0). ΔDCP-VD (percentage points; T1 − T0) in the remission (CR/CRi, n = 54; range 0.0 to +3.5) versus nonremission (PR/NR, n = 23; range −0.5 to +2.5) group: 1.50 (1.00, 2.00) vs. 0.50 ± 0.83 percentage points; Mann–Whitney U *p* < 0.01; Hedges g = 1.20 (95% CI 0.68–1.72). DCP-VD, deep capillary plexus vessel density; CR, complete remission; CRi, complete remission with incomplete hematologic recovery; PR, partial remission; NR, no response.

At T1, 154 eye scans from 77 patients passed quality control; 143 of these had a quality-eligible T0 scan of the same eye and were analyzed under the same-eye pairing rule (66 patients contributing both eyes, 11 contributing one eye), and the remaining 11 eyes lacked an eligible T0 counterpart. ΔDCP-VD differed significantly between response groups (Table 4, Figure 4): 1.50 (1.00, 2.00) percentage points in the remission group (CR/CRi, n = 54; range 0.0 to +3.5) versus 0.50 ± 0.83 percentage points in the nonremission group (PR/NR, n = 23; range −0.5 to +2.5), a between-group difference of 0.97 percentage points (95% CI 0.57–1.37; Mann–Whitney U *p* < 0.01; Welch t = 4.787, *p* < 0.01; Hedges g = 1.20, 95% CI 0.68–1.72). In a sensitivity analysis including the focal outcome together with the 12 longitudinal secondary outcomes in a single Benjamini–Hochberg family (13 tests), the focal comparison remained significant (q < 0.01). Nine secondary outcomes (ΔIRVD, ΔSCP-VD, ΔDCP-FAZ area, ΔVDCC, Δ3D-CVI, ΔCVV/a, ΔDCP-VLD, ΔDCP-FD, and ΔDCP junction density) also differed between groups and survived FDR correction (all q < 0.01), whereas ΔRPC-VD, Δwhole-FAZ area, and ΔVDMLCV did not (q range 0.092–0.722; Table 4). By T1, patient-level retinal hemorrhage prevalence had fallen from 19.8% at baseline to 9.1% (7/77); among the 17 completers with baseline hemorrhage, it had resolved in 10 and persisted in 7, and no new hemorrhages appeared (exact McNemar *p* < 0.01, denominator 77; exploratory, without multiplicity adjustment). Image quality remained high and stable (T0 mean IQ 8.90 ± 1.09, median 9 (IQR 8–10) across 283 analyzed eyes; T1 mean 8.45 ± 1.08, median 8 (IQR 8–9); patient-level ΔIQ among completers −0.24 ± 1.51, *p* = 0.198), with no difference between response groups (ΔIQ 0.00 (−1.38, 1.00) vs. −0.46 ± 1.54, *p* = 0.353), arguing against quality drift as an explanation for the response-group ΔDCP-VD difference. The response-group difference was concordant across approaches to fellow-eye data (eye-level cluster-robust model and linear mixed-effects model, both B = +1.02; both-eyes-eligible subset +1.08; Section 3.6). The concurrent DCP-FAZ changes in the remission group—enlarging area (+0.04 mm^2^) with shortening perimeter (−0.11 mm)—indicate a more circular, less irregular FAZ contour after induction rather than a simple expansion of avascular retina; FAZ segmentation followed the identical device pipeline and reader-review rules at both time points, although improved flow-signal detection at T1 can itself shift the automated FAZ boundary (Section 4.5).

**Table 4:** Longitudinal changes in OCTA metrics after first induction therapy by response group (same-eye pairing).

Δ Metric (T1 − T0)	Remission, CR/CRi (n = 54)	Nonremission, PR/NR (n = 23)	*p* Value	q Value
ΔDCP-VD, percentage points (main exploratory outcome)	+1.50 (1.00, 2.00)	+0.50 ± 0.83	<0.01	NA
ΔSCP-VD, percentage points	+0.00 (−0.50, 0.00)	−0.98 ± 1.06	<0.01	<0.01
ΔIRVD, percentage points	+1.63 (1.16, 2.00)	+0.00 ± 0.75	<0.01	<0.01
ΔRPC-VD, percentage points	+0.00 (−0.50, 0.50)	+0.00 (−0.75, 0.50)	0.722	0.722
† ΔSCP-FAZ area, mm^2^	−0.01 ± 0.02	−0.00 ± 0.02	0.545	0.654
ΔDCP-FAZ area, mm^2^	+0.04 ± 0.03	+0.01 ± 0.04	<0.01	<0.01
ΔWhole-FAZ area, mm^2^	−0.01 ± 0.02	−0.01 (−0.03, −0.00)	0.076	0.092
† ΔSCP-FAZ perimeter, mm	+0.00 (−0.05, +0.06)	+0.00 (−0.08, +0.02)	0.139	0.208
† ΔDCP-FAZ perimeter, mm	−0.11 (−0.15, −0.02)	−0.02 (−0.07, +0.19)	<0.01	<0.01
† ΔWhole-FAZ perimeter, mm	−0.05 ± 0.07	−0.05 ± 0.07	0.868	0.868
ΔVDCC, percentage points	+1.00 (+0.50, +1.50)	−1.00 (−1.50, −1.00)	<0.01	<0.01
ΔVDMLCV, percentage points	−4.00 (−5.50, −3.00)	−5.00 (−5.50, −3.00)	0.650	0.709
Δ3D-CVI, percentage points	+3.50 (+3.00, +4.00)	+2.00 (+1.75, +2.50)	<0.01	<0.01
ΔCVV/a, μm	+5.75 (+4.50, +7.00)	+4.07 ± 1.58	<0.01	<0.01
† ΔCSV/a, μm	−10.50 (−15.38, −8.50)	−5.43 ± 3.07	<0.01	<0.01
† ΔSFCT, μm	−10.06 ± 10.70	+9.89 ± 6.10	<0.01	<0.01
ΔDCP-VLD, mm/mm^2^	+0.30 (+0.10, +0.42)	−0.23 ± 0.30	<0.01	<0.01
ΔDCP-FD	+0.06 (+0.05, +0.09)	+0.00 ± 0.03	<0.01	<0.01
ΔDCP junction density, n/mm^2^	+6.14 ± 1.83	+2.50 ± 0.83	<0.01	<0.01

Data are mean ± SD or median (IQR), as appropriate to Shapiro–Wilk normality; between-group tests were independent-samples *t* tests or Mann–Whitney U tests accordingly. q values: Benjamini–Hochberg FDR within the 12-outcome SAP-designated secondary family (nine outcomes—ΔIRVD, ΔSCP-VD, ΔDCP-FAZ area, ΔVDCC, Δ3D-CVI, ΔCVV/a, ΔDCP-VLD, ΔDCP-FD, and ΔDCP junction density—survived correction, all q < 0.01) and, for the six rows marked †, within a separate 6-test exploratory family (ΔSFCT, ΔDCP-FAZ perimeter, and ΔCSV/a survived correction, all q < 0.01). The focal outcome was not part of the 12-outcome family (q not applicable, shown as NA); in a 13-test sensitivity family including the focal outcome, its q was < 0.01. Main exploratory outcome: between-group difference 0.97 percentage points (95% CI 0.57–1.37), Mann–Whitney U *p* < 0.01, Hedges g = 1.20 (95% CI 0.68–1.72). Δ denotes T1 minus T0; VD, vessel density; IRVD, inner retinal vessel density; SCP, superficial capillary plexus; DCP, deep capillary plexus; RPC, radial peripapillary capillaries; FAZ, foveal avascular zone; VDCC, choriocapillaris vessel density; VDMLCV, medium/large choroidal vessel density; 3D-CVI, three-dimensional choroidal vascularity index; CVV/a, choroidal vessel volume per unit area; VLD, vessel length density; FD, fractal dimension; CR, complete remission; CRi, CR with incomplete hematologic recovery; PR, partial remission; NR, no response; IQR, interquartile range; *p* values are reported to three decimal places (values below 0.01 are denoted as *p* < 0.01); no asterisk annotation is used.

### 3.4. Correlates of Microvascular Change

**Figure 5:**
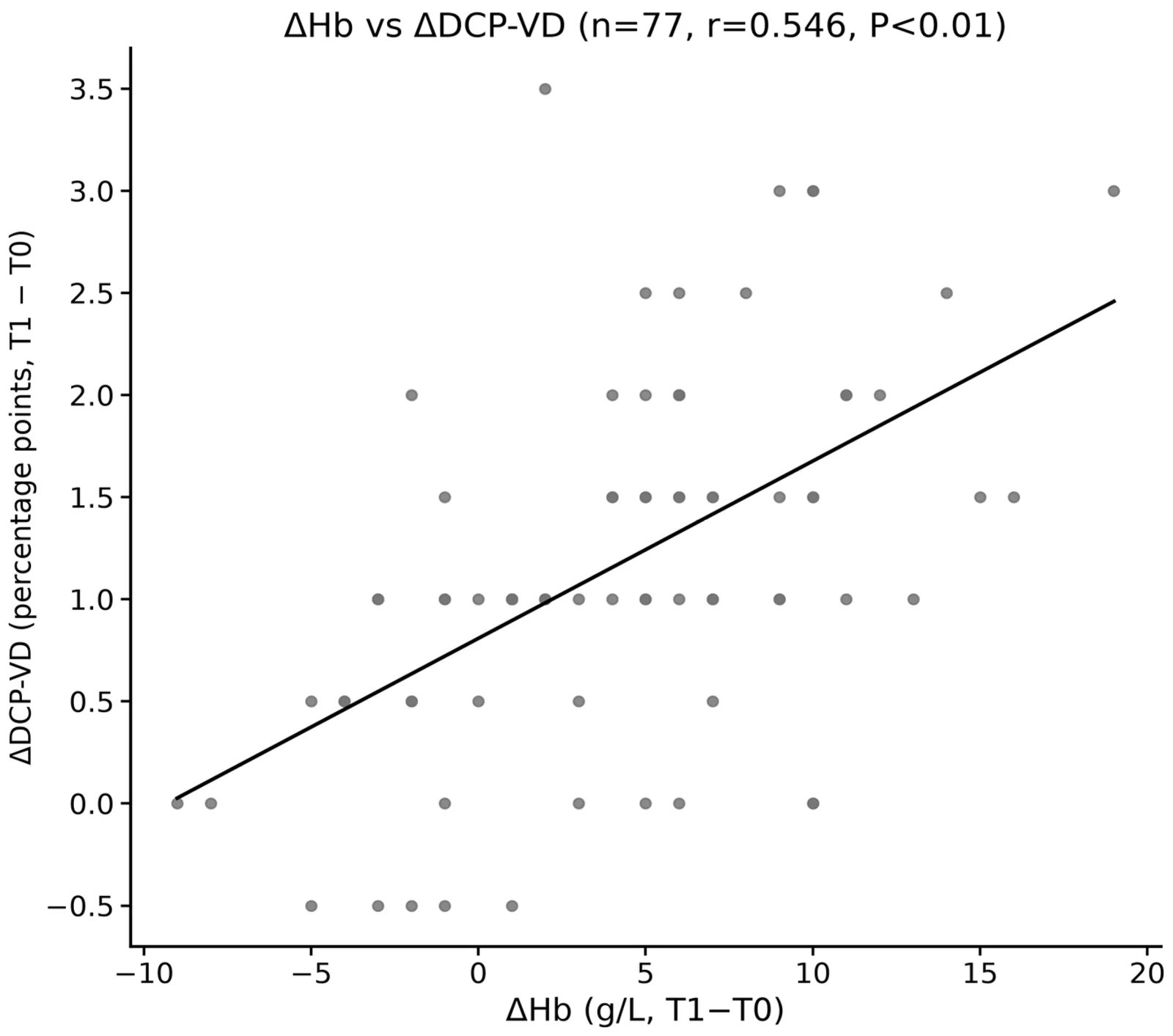
Association between hemoglobin change and DCP-VD change. Scatter plot of patient-level ΔHb versus ΔDCP-VD (both T1 − T0; ΔDCP-VD in percentage points) with the unadjusted ordinary least squares fit (n = 77). Spearman r_s_ = +0.546 (95% CI 0.367–0.686); *p* < 0.01; Benjamini–Hochberg q < 0.01 within the five-correlation family. ΔHb, change in hemoglobin; DCP-VD, deep capillary plexus vessel density.

During induction, Hb rose by 4.30 ± 5.63 g/L on average and bone marrow blasts fell by 22.06 ± 7.86%. Because ΔDCP-VD was non-normally distributed, correlations were computed with the Spearman method. ΔHb correlated most strongly with ΔDCP-VD (r_s_ = +0.546, 95% CI 0.367–0.686, *p* < 0.01, Benjamini–Hochberg q < 0.01 within the five-correlation family; Figure 5). ΔPLT showed a weak association (r_s_ = +0.334, *p* < 0.01) that remained significant after correction (q < 0.01); ΔWBC (r_s_ = −0.034, *p* = 0.770), Δperipheral blasts (r_s_ = +0.078, *p* = 0.500), and Δbone marrow blasts (r_s_ = −0.068, *p* = 0.556) showed no significant association. The ΔHb-ΔDCP-VD association was directionally consistent within both response groups (CR/CRi r_s_ = +0.409, *p* < 0.01; PR/NR r_s_ = +0.464, *p* = 0.026) and persisted after adjustment for response status (rank-based partial r = +0.447, *p* < 0.01). In multivariable regression (n = 77; R^2^ = 0.355; all variance inflation factors < 1.15), ΔHb remained positively associated with ΔDCP-VD after adjustment (B = 0.086 per g/L, 95% CI 0.053–0.119, *p* < 0.01), whereas platelet transfusion volume was negatively associated (B = −0.119, 95% CI −0.217 to −0.021, *p* = 0.018)—an association consistent with confounding by indication, as transfusion volume tracked lower baseline PLT (r_s_ = −0.446, *p* < 0.01, n = 111) (Table 5).

**Table 5:** Multivariable linear regression of factors associated with ΔDCP-VD (n = 77).

Variable	B	95% CI	*p* Value	VIF
ΔHb, g/L	0.086	0.053 to 0.119	<0.01	1.14
Δbone marrow blasts, %	−0.002	−0.024 to +0.020	0.856	1.03
RBC transfusion, U	−0.013	−0.083 to +0.058	0.725	1.07
Platelet transfusion, doses	−0.119	−0.217 to −0.021	0.018	1.08
First-course anthracycline, mg/m^2^	0.001	−0.002 to +0.004	0.591	1.15

Model R^2^ = 0.355, adjusted R^2^ = 0.309; overall F(5,71) = 7.81, *p* < 0.01. Univariable correlations with ΔDCP-VD (all Spearman): ΔHb r_s_ = +0.546 (*p* < 0.01, q < 0.01); ΔPLT r_s_ = +0.334 (*p* < 0.01, q < 0.01); ΔWBC r_s_ = −0.034 (*p* = 0.770); Δperipheral blasts r_s_ = +0.078 (*p* = 0.500); Δbone marrow blasts r_s_ = −0.068 (*p* = 0.556); q values from Benjamini–Hochberg correction across the five-correlation family. The negative platelet-transfusion association is interpreted as confounding by indication (transfusion volume inversely correlated with baseline PLT, r_s_ = −0.446, *p* < 0.01, n = 111). Model diagnostics: residual Shapiro–Wilk *p* = 0.270; Breusch-Pagan *p* = 0.838; maximum VIF = 1.15. Seven observations exceeded the influence threshold (Cook’s distance > 4/n, maximum 0.094); these observations were retained, and deleting them did not change the direction of the ΔHb or platelet-transfusion coefficients. When response status was added to the model (R^2^ = 0.434), ΔHb remained significantly associated with ΔDCP-VD (B = 0.059, *p* < 0.01), whereas the platelet-transfusion coefficient was attenuated and no longer significant (B = −0.055, *p* = 0.283), consistent with a confounded, non-independent association rather than a stable independent effect; response status itself remained strongly associated (B = +0.768, *p* < 0.01). VIF, variance inflation factor; *p* values are reported to three decimal places (values below 0.01 are denoted as *p* < 0.01); no asterisk annotation is used.

### 3.5. Exploratory Discrimination Performance of Δdcp-Vd

**Figure 6:**
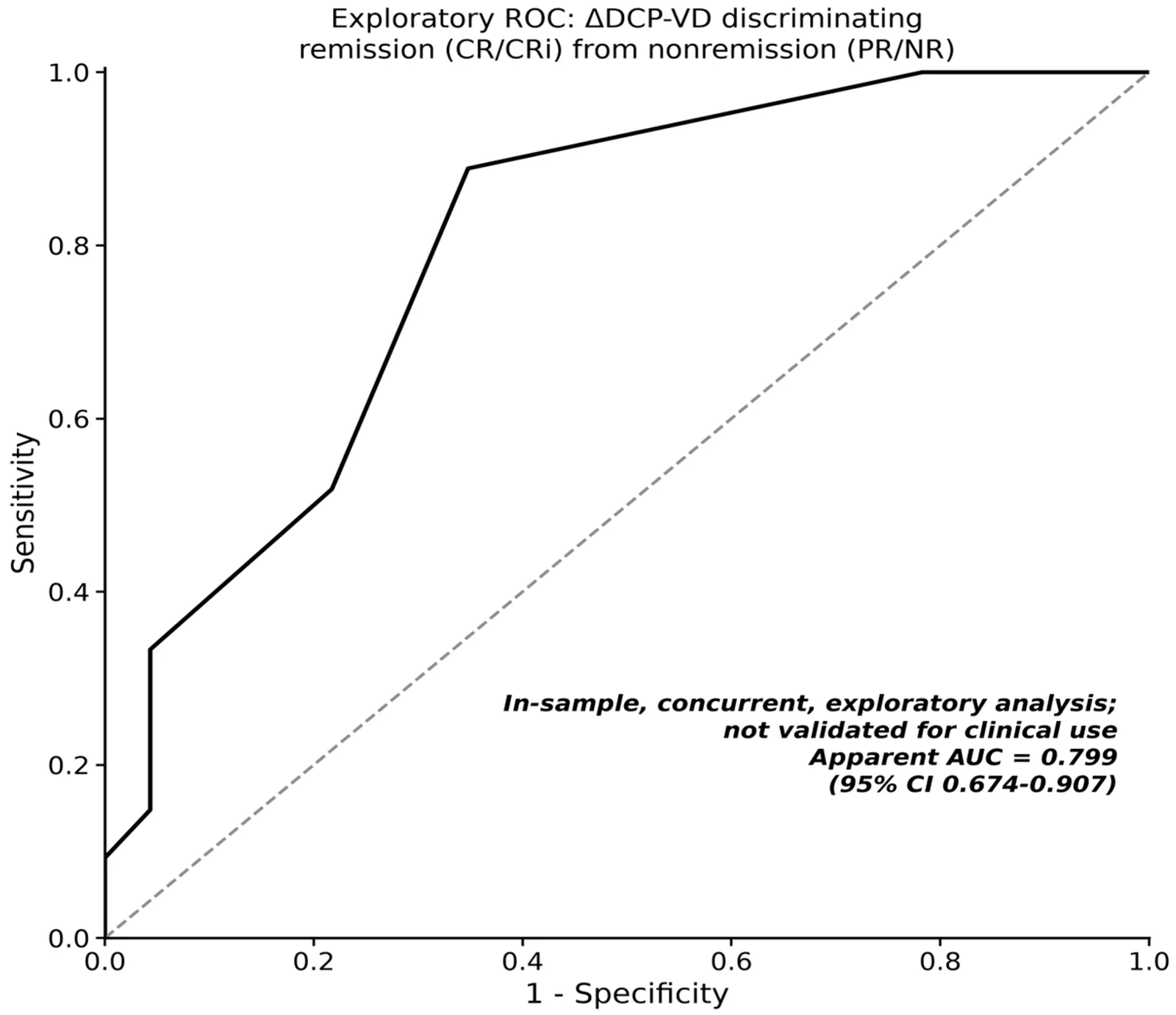
Exploratory receiver operating characteristic (ROC) analysis of ΔDCP-VD (percentage points; T1 − T0) for discriminating remission (CR/CRi, n = 54) from nonremission (PR/NR, n = 23) at the patient level. Apparent, patient-level, in-sample area under the curve (AUC) 0.799 (bootstrap 95% CI 0.674–0.907; 5000 resamples); hypothesis-generating estimates of exploratory discrimination performance; post hoc sample-specific, descriptive operating characteristics in Table S6. Imaging and response were assessed concurrently at T1; the analysis reflects association, not prediction. ΔDCP-VD, change in deep capillary plexus vessel density; CR, complete remission; CRi, complete remission with incomplete hematologic recovery; PR, partial remission; NR, no response.

A post hoc exploratory ROC analysis showed apparent in-sample separation between the remission (CR/CRi) and nonremission (PR/NR) groups by ΔDCP-VD, with an apparent AUC of 0.799 (bootstrap 95% CI 0.674–0.907; Figure 6; Table S6 and the Supplementary Statistical Note). Because imaging and response were assessed concurrently at T1, these estimates describe an association between microvascular change and treatment response, not prediction of remission. The AUC is an apparent, patient-level, in-sample estimate with a bootstrap 95% CI; neither the AUC nor the post hoc sample-specific Youden point and its operating characteristics (Table S6; descriptive only, not a clinical cutoff) have been internally or externally validated; the bootstrap confidence interval quantifies sampling variability of the apparent AUC only and does not correct for selection optimism. These estimates are hypothesis-generating only.

### 3.6. Sensitivity Analyses

The T0-to-T1 interval correlated weakly with ΔDCP-VD (Spearman r_s_ = +0.251, *p* = 0.028), and the ≤42-day (n = 40) versus >42-day (n = 37) subgroups differed nominally (1.00 (0.38, 1.50) vs. 1.39 ± 0.83 percentage points, *p* = 0.036); after adjustment for response status, the interval was not independently associated (B = +0.019/day, *p* = 0.103), whereas remission status remained strongly associated (B = +0.935, *p* < 0.01). In ANCOVA addressing change-baseline coupling (R^2^ = 0.950), both remission status (B = +0.56, 95% CI 0.16–0.97, *p* < 0.01) and ΔHb (B = +0.069, 95% CI 0.036–0.103, *p* < 0.01) retained adjusted associations; the ΔHb coefficient is a conditional association and should not be interpreted as a confounding-adjusted total group difference or as evidence of mediation. An eye-level longitudinal change-score model with patient-level cluster-robust standard errors confirmed the response-group difference (B = +1.02, 95% CI 0.65–1.39, *p* < 0.01); a linear mixed-effects model (286 eye-time observations from 143 same-eye pairs; patient random intercept, maximum-likelihood estimation) gave a consistent time × response interaction (B = +1.02, 95% CI 0.20–1.84, *p* = 0.015). Among the 92 patients with two eligible eyes at T0, the fellow-eye ICC(2,1) was 0.819 for IRVD (95% CI 0.743–0.879) and 0.585 for DCP-VD (0.435–0.706); the pooled ICC across patients and controls (n = 132 pairs; 0.847 and 0.607) was higher for IRVD because of between-group heterogeneity and is not interpreted as test–retest or inter-reader reliability. Hemorrhage grade correlated inversely with DCP-VD (patient-level Spearman r_s_ = −0.280, *p* < 0.01; eye-level analysis of the 203 quality-eligible eyes r_s_ = −0.236, *p* < 0.01; patient-level cluster bootstrap over 2000 resamples, 91.7% of resamples *p* < 0.05) (Table S5). Excluding eyes with baseline retinal hemorrhage (n = 60; CR/CRi 42, PR/NR 18) likewise gave a consistent between-group difference (0.96 percentage points; +1.43 vs. +0.47, *p* < 0.01; Table S5), indicating that the response-group difference is not driven by hemorrhage-related signal attenuation. The four-category response distribution was consistent with the composite grouping (Kruskal–Wallis H = 21.522, *p* < 0.01, rank effect size ε^2^ = 0.254): ΔDCP-VD was positive in both CR (1.66 ± 0.72, n = 25) and CRi (1.00 (1.00, 1.50), n = 29; Bonferroni-adjusted between-subgroup *p* = 0.240) and near zero in PR (0.41 ± 0.71, n = 16) and NR (0.71 ± 1.07, n = 7; Bonferroni-adjusted *p* = 1.000); CR/CRi versus PR subgroup comparisons were significant (CR vs. PR *p* < 0.01; CRi vs. PR *p* < 0.01), whereas CR vs. NR (*p* = 0.156) and CRi vs. NR (*p* = 0.788) were not, supporting the composite grouping (Table S5). Within-group paired changes were significant in CR (43.62% → 45.28%, paired t = 11.57, *p* < 0.01) and CRi (42.00% → 43.31%, Wilcoxon *p* < 0.01), small in PR (42.69% → 43.09%, paired t = 2.28, *p* = 0.038), and absent in NR (43.57% → 44.29%, *p* = 0.129).

Post hoc attrition sensitivity analyses (August 2026 analysis-plan revision) supported the robustness of the main comparison. Inverse probability weighting (completion-model C statistic 0.665; stabilized weights 0.76–1.63 after 1st/99th-percentile truncation; effective sample size 73.9) yielded a weighted between-group difference of +1.04 percentage points (95% CI 0.66–1.42, *p* < 0.01); multiple imputation under a missing-at-random assumption gave +0.96 (95% CI 0.55–1.37); and the δ-tipping analysis showed that the main comparison would lose statistical significance only if non-completers who would have achieved remission had ΔDCP-VD systematically at least 1.75 percentage points lower than same-subtype completers (δ* = −1.75), a shift about 1.8-fold the observed between-group difference. A Welch-corrected test accounting for unequal variances gave a consistent result (t = 4.787, *p* < 0.01), as did an extended covariance model additionally adjusting for subtype, follow-up interval, and ΔIQ (remission B = +0.54, 95% CI 0.13–0.94, *p* = 0.011) and an analysis restricted to the 66 patients with both eyes eligible (+1.08, 95% CI 0.67–1.48, *p* < 0.01). These analyses rest on measured baseline variables and cannot exclude bias from unmeasured factors.

## 4. Discussion

### 4.1. Principal Findings

In this prospective longitudinal cohort of adults with newly diagnosed acute leukemia imaged with 16 × 16 mm WF-SS-OCTA, retinal and choroidal microvascular metrics at diagnosis differed between patients and healthy controls—11 of 20 metrics reached nominal significance and 10 survived FDR correction—while the DCP-VD gradient (AML < ALL < control) was not explained by age, sex, or baseline hemoglobin in adjusted models; retinal hemorrhage, though infrequent and mild, tracked baseline Hb and DCP-VD. After the first induction therapy, ΔDCP-VD was positive in patients achieving composite remission (CR/CRi) and near zero in those without remission (PR/NR), accompanied by concordant response-group differences in retinal and choroidal density metrics and skeletonized morphology, and the magnitude of change was associated with the concurrent hemoglobin increase after adjustment for treatment exposures. The negative association between platelet transfusion volume and ΔDCP-VD likely reflects disease severity and baseline thrombocytopenia rather than a direct biological inhibitory effect of transfusion. Exploratory analysis further showed apparent in-sample separation of remission status by ΔDCP-VD (AUC 0.799), an estimate that is optimistic by construction and requires external validation. Because the focal outcome was designated after data collection, all of these findings are hypothesis-generating and define questions for confirmatory studies rather than constituting confirmatory evidence. These findings suggest that WF-SS-OCTA may provide a noninvasive, research-stage approach for evaluating microvascular alterations and treatment-associated change during acute leukemia therapy.

### 4.2. Biological Interpretation of Dcp-Vd

DCP-VD was designated the main exploratory imaging outcome after completion of data collection because it was the only retinal vessel-density metric showing an ordered AML < ALL < healthy-control gradient with a nominally significant baseline between-group difference (η^2^ = 0.056), and because the DCP represents a retinal vascular compartment that may be particularly sensitive to perfusion alterations.

The preferential involvement of the DCP may be explained by its anatomical and metabolic characteristics: this plexus perfuses the highly metabolic inner nuclear layer at the watershed between the retinal and choroidal oxygen supplies, where oxygen tension is low and perfusion reserve is thin, rendering it vulnerable to hypoxia, stasis, and perfusion pressure fluctuations [18,19]. Under the anemic, hyperviscous, and inflammatory milieu of acute leukemia, DCP perfusion plausibly falls below the OCTA flow-detection threshold earliest and most extensively. Several mechanisms may contribute to DCP vulnerability in acute leukemia: anemia-related hypoxia, leukostasis, and endothelial activation may reduce perfusion reserve and impair capillary flow, particularly in metabolically demanding retinal layers. These mechanistic interpretations are hypotheses that were not directly tested in this study. Our data are consistent with this framework: baseline Hb correlated with SFCT and inversely with hemorrhage; ΔHb was the strongest correlate of the longitudinal change; and the literature documents that leukemic blasts activate endothelium and secrete interleukin-6, tumor necrosis factor-alpha, and vascular endothelial growth factor, suggesting an interaction between inflammation, endothelial activation, and microvascular impairment [41,42,43,44,45,46,47,48,49].

Two cautions are essential. First, DCP sensitivity is not leukemia-specific: similar DCP-predominant loss occurs in iron-deficiency anemia and sickle cell disease [50,51,52,53,54], so DCP-VD is best regarded as a sensitive but non-specific gauge of microcirculatory stress whose disease attribution requires clinical context. Second, OCTA measures flow signal, not capillary counts; the observed changes likely mix functional hypoperfusion with structural dropout, and the rapid post-induction increase in DCP-VD among patients achieving remission is consistent with a substantial functional component —and, in this cohort, the flow-signal increase was paralleled by response-group differences in skeletonized morphology metrics (vessel length density, fractal dimension, and junction density), indicating concomitant network-level change; taken together, these findings are compatible with, but do not establish, a temporal sequence or a structural-versus-functional partition of the post-induction recovery. The AML-below-ALL numerical gradient did not reach statistical significance after Bonferroni adjustment (*p* = 0.294) or covariate adjustment (B = −0.782, *p* = 0.176) and should therefore not be interpreted as an intrinsic subtype effect; residual confounding by age structure, molecular characteristics, leukocyte burden, and treatment intensity cannot be excluded.

### 4.3. Research Implications and Marker-Development Considerations

**Figure 7:**
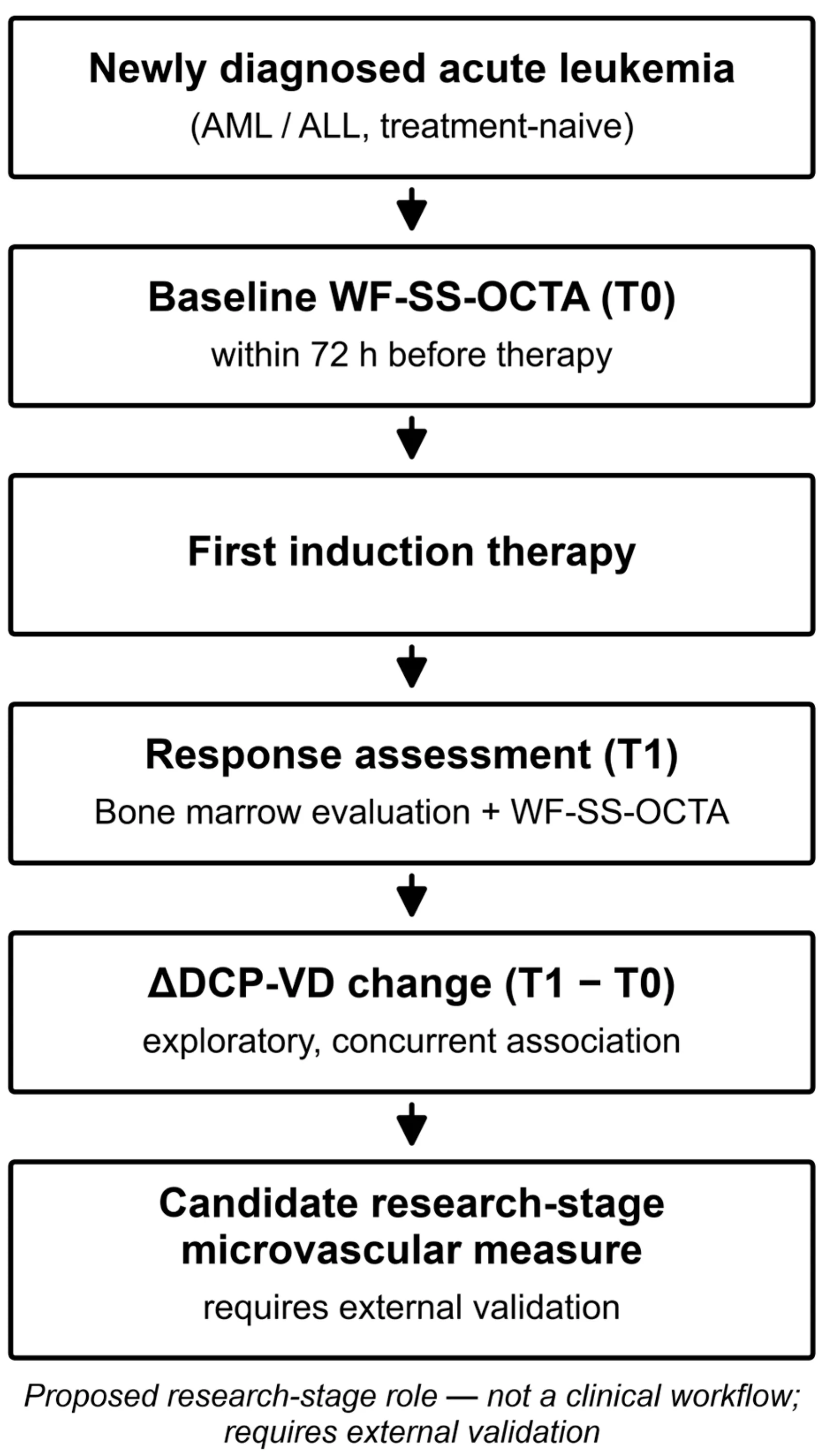
Proposed research framework for WF-SS-OCTA in acute leukemia treatment evaluation. At diagnosis, baseline WF-SS-OCTA (T0) characterizes leukemia-associated microvascular alterations; after induction therapy, T1 imaging is performed alongside bone marrow response assessment, and ΔDCP-VD provides a quantitative, research-stage measure of microvascular change associated with the concurrent response assessment. This pathway is a hypothetical, research-stage complement to—not a replacement for—marrow-based response assessment and requires external validation before any clinical application. DCP-VD, deep capillary plexus vessel density; WF-SS-OCTA, wide-field swept-source optical coherence tomography angiography.

Bone marrow examination remains the reference standard for response assessment, but it is invasive, impractical at high frequency, and may be deferred in the setting of severe thrombocytopenia or poor tolerance. DCP-VD should not replace bone marrow response assessment, but may provide complementary information regarding microvascular change. The present findings offer preliminary support, conceptually aligned with the FDA-NIH BEST framework [55]: biological plausibility, layered image-quality control, and association with hematologic response. Hypothetical research-stage applications (Figure 7), all requiring prospective validation, include microvascular assessment alongside scheduled response evaluations and studies of treatment-related vascular toxicity, such as cumulative anthracycline endothelial injury and transplantation-associated thrombotic microangiopathy [48,49,56,57]. Because imaging and response were assessed concurrently at T1, ΔDCP-VD describes the microvascular state associated with the response assessment rather than predicting it; longitudinal sampling during therapy would be needed to evaluate any early-warning value.

Equally important are the limits: the ROC estimates were derived in-sample and are optimistic by construction; no validated ΔDCP-VD threshold exists; cross-device comparability is unresolved; and the present data do not demonstrate incremental value of ΔDCP-VD over routine hemoglobin and blood-count indices or its specificity relative to non-leukemic anemia, so statistical separation does not imply clinical utility. DCP-VD should therefore be viewed as a research-stage candidate marker requiring multicenter external validation, threshold definition, and prospective utility studies. This observational cohort was reported according to STROBE; the post hoc ROC analysis was a single-marker, concurrent exploratory analysis rather than a diagnostic-accuracy study, so a complete STARD checklist was not applied, and its principles were used only to frame the risks of post hoc marker selection, same-sample performance estimation, data-derived operating-point selection, and absence of validation [58]. No multivariable prediction model was developed, so TRIPOD and PROBAST were not formally applicable [59,60]; any future threshold-based or prediction-model studies should follow that guidance. The standardized acquisition and data-management pipeline established here offers reusable infrastructure for such validation and for future machine-learning models [61,62,63].

### 4.4. Comparison with Previous Studies

Our baseline hemorrhage prevalence (19.8%) is lower than the 35–64% ocular-involvement rates in classic series [6,7,8], plausibly because we imaged patients within 72 h before therapy, required fixation capacity for wide-field scanning (enriching for clinically stable patients), and applied a hemorrhage-centered sign definition; the direction of association with Hb nonetheless matched those reports. Relative to prior leukemia OCTA studies [20,21,22,23], to our knowledge, based on a non-systematic search of PubMed and Google Scholar (terms combining acute leukemia and optical coherence tomography angiography) through July 2026, this cohort is among the largest (111 patients) and among the first to include parallel AML and ALL arms, to add skeletonized DCP morphology, and to anchor same-eye pre/post-induction change to adjudicated response; an ultra-widefield OCTA study in remission-phase acute leukemia has recently been reported by Xu et al. [24], and our 16 × 16 mm pre/post-induction design complements that work. Cicinelli et al. reported a numerically lower baseline SCP density (*p* = 0.08) and post-remission microvascular changes in 12 patients [20]; Yang et al. reported partially reversible macular deficits with choroidal thickening in 48 AML patients [21]; Zhou et al. found persistent DCP deficits in remission-phase patients without retinopathy [22]. We extend these observations by quantifying the response-group separation of microvascular change and by identifying the concurrent hemoglobin increase as its strongest correlate. Notably, our cross-sectional patient-versus-control differences were broad but moderate in magnitude (11 of 20 metrics nominally significant, 10 surviving FDR correction), with absolute between-group differences smaller than the large deficits reported in earlier small macula-centered series and no AML-versus-ALL difference after covariate adjustment; differences in devices, scanning fields, disease phase, and analysis populations likely contribute to this heterogeneity. SFCT differed across groups in our cohort (*p* = 0.019; AML lower than ALL, *p* = 0.037), in contrast to the infiltrative thickening reported by Yang et al. and in case reports; SFCT correlated positively with baseline Hb (r_s_ = +0.217, *p* = 0.022) and CVV/a (r_s_ = +0.723, *p* < 0.01), and negatively with age (r_s_ = −0.318, *p* < 0.01), suggesting that choroidal thickness in this cohort is modulated by anemia-related perfusion status rather than by clinically visible infiltration (3/111 patients). Cross-study comparison of absolute vessel-density values is further limited by differences in devices, scanning fields, slab definitions, and magnification correction.

### 4.5. Limitations

This study has several limitations. First, it is a single-center study with a modest sample—particularly 23 nonremission patients—and 30.6% attrition; although no baseline variable differed significantly between completers and non-completers (absolute standardized mean differences up to 0.32), missingness may be informative (9 progression-related non-completions), and severely ill patients unable to fixate were likely underrepresented, biasing prevalence and severity estimates toward the mild end, and external validity requires multicenter confirmation. Second, the observational design precludes causal inference: the ΔHb-ΔDCP-VD association may be jointly driven by remission or resolving inflammation; because both variables change with treatment response, ΔHb may act as a confounder, a mediator, or a co-outcome of microvascular change, and the adjusted association (rank-based partial r = +0.447) cannot distinguish among these roles. No direct oximetry, viscosity, or endothelial biomarker measurements were available. Third, the statistical analysis plan was finalized after completion of data collection, and the trial registration did not define an imaging primary outcome; although the main comparison was designated before the response-association analyses were run, all results—particularly the ROC analysis—should be regarded as exploratory and hypothesis-generating. Fourth, T1 was a single assessment within a 28–56-day window; longer-term trajectories and prognostic value are unknown. The T1 scan preceded or followed the response-assessment marrow examination by up to 7 days (median 2 days), and analyses restricted to narrower imaging-to-marrow windows were not performed. Fifth, measurement constraints apply: morphology was quantified over the full 16 × 16 mm field without peripheral subfield analysis; device metrics and the Fiji pipeline are not cross-platform portable; test–retest repeatability of this 16 × 16 mm protocol and inter-reader repeatability of the manual masking steps were not formally assessed; the observed between-group difference (0.97 percentage points) is of the same order as the device export granularity (integer vessel-density percentages; 0.5-unit patient-level increments), so ΔDCP-VD can at present be interpreted only at the group level, and repeatability coefficients and minimal detectable change for this wide-field protocol must be established before any individual-patient interpretation; and the acquisition/analysis manual was formally consolidated after data collection, so blinding documentation is incomplete, although the underlying data are unaffected. Sixth, cross-sectional comparisons are between-person contrasts that remain sensitive to sampling variability and residual confounding: although 10 of 20 baseline metrics survived multiplicity correction, the AML-versus-ALL gradient did not persist after covariate adjustment, so the cross-sectional findings should be read as exploratory, and the study’s main inferences rest on the longitudinal, within-person response contrast. Fellow-eye ICC values (IRVD 0.819; DCP-VD 0.585) quantify between-eye biological concordance only and do not substitute for test–retest or inter-reader reliability; flow-signal metrics may reflect perfusion state rather than fixed capillary anatomy, and device- or algorithm-specific values are not directly interchangeable across platforms. Seventh, the timing of red-cell and platelet transfusions relative to the T0 and T1 hemoglobin draws was not recorded; because transfusion acutely raises hemoglobin, ΔHb values carry timing-related variability that cannot be quantified. Eighth, healthy controls underwent a single examination, so systematic drift (device, technician, or seasonal) cannot be formally excluded as a contributor to within-person change, although the stable image-quality scores argue against gross quality drift. Ninth, the NR subgroup comprised only 7 patients, so the nonremission contrast is dominated by PR; visual-function and optical-quality correlates were not collected; and the absence of a non-leukemic anemia control group limits the specificity of the microvascular findings for acute leukemia.

## 5. Conclusions

In this selected single-center cohort, adults with newly diagnosed acute leukemia showed quantifiable retinal and choroidal microvascular alterations at diagnosis—most consistently in AML and not attributable to age, sex, or baseline hemoglobin—together with an ordered, exploratory DCP-VD gradient across AML, ALL, and healthy controls. Among the 77 patients completing same-eye paired imaging, ΔDCP-VD differed clearly by concurrent morphologic/hematologic response category and was associated with the concurrent hemoglobin increase after adjustment for treatment exposures. Because the focal outcome was designated after data collection, these findings are hypothesis-generating: they do not establish causal treatment effects, diagnostic accuracy, prediction, or monitoring utility; ΔDCP-VD should be regarded as a research-stage imaging measure of treatment-associated microvascular change, and no data-derived threshold should be used clinically before confirmation in a prospectively specified, independently collected cohort and across OCTA platforms.

## Data Availability

De-identified patient-level data and the analysis code that generates all tables and figures are available from the corresponding author upon reasonable request, subject to approval by the Biomedical Ethics Committee of West China Hospital and a data-sharing agreement, because the data contain potentially identifiable clinical information. The image-quality inclusion status of each eye and the derived patient-level dataset underlying all analyses can be shared in the same manner.

## Supplementary Materials

The following supporting information can be downloaded at: https://www.mdpi.com/article/10.3390/diagnostics16182940/s1, Supplementary Method S1: Wide-Field SS-OCTA Acquisition Standard Operating Procedure; Supplementary Method S2: Image Quality Control; Supplementary Method S3: Fiji/ImageJ Deep Capillary Plexus Morphology Pipeline; Supplementary Method S4: Choroidal Quantification; Table S1: Fundus manifestations at diagnosis; Table S2: Full FAZ metrics; Table S3: Full choroidal and structural OCT metrics; Table S4: Full DCP morphology metrics; Table S5: Sensitivity and supplementary analyses; Table S6: Exploratory ROC analysis details; Table S7: Treatment exposures during the T0–T1 interval by subtype; Figure S1: Representative fundus manifestations at T0; Supplementary Statistical Note: Exploratory discrimination analysis.

## Abbreviations

3D-CVI, three-dimensional choroidal vascularity index; ALL, acute lymphoblastic leukemia; AML, acute myeloid leukemia; ANCOVA, analysis of covariance; AUC, area under the curve; BCVA, best-corrected visual acuity; CI, confidence interval; CMT, central macular thickness; CR, complete remission; CRi, complete remission with incomplete hematologic recovery; CSV/a, choroidal stromal volume per unit area; CVV/a, choroidal vessel volume per unit area; DCP, deep capillary plexus; FAZ, foveal avascular zone; FD, fractal dimension; FDR, false discovery rate; Hb, hemoglobin; HC, healthy control; ICC, intraclass correlation coefficient; IOP, intraocular pressure; IPW, inverse probability weighting; IQ, image quality; IQR, interquartile range; IRVD, inner retinal vessel density; LMM, linear mixed-effects model; MICE, multiple imputation by chained equations; MRD, measurable residual disease; NR, no response; OCTA, optical coherence tomography angiography; PLT, platelet count; PR, partial remission; RBC, red blood cell; ROC, receiver operating characteristic; RPC, radial peripapillary capillaries; SAP, statistical analysis plan; SCP, superficial capillary plexus; SD, standard deviation; SFCT, subfoveal choroidal thickness; SMD, standardized mean difference; VD, vessel density; VDCC, choriocapillaris vessel density; VDMLCV, medium/large choroidal vessel density; VIF, variance inflation factor; VLD, vessel length density; WBC, white blood cell count; WF-SS-OCTA, wide-field swept-source optical coherence tomography angiography; ΔDCP-VD, change in deep capillary plexus vessel density.

## References

[1] Porcu P., Cripe L.D., Ng E.W., Bhatia S., Danielson C.M., Orazi A., McCarthy L.J. (2000). Hyperleukocytic Leukemias and Leukostasis: A Review of Pathophysiology, Clinical Presentation and Management. Leuk. Lymphoma.

[2] Kwaan H., Bongu A. (1999). The Hyperviscosity Syndromes. Semin. Thromb. Hemost..

[3] Patton N., Aslam T.M., MacGillivray T., Deary I.J., Dhillon B., Eikelboom R.H., Yogesan K., Constable I.J. (2006). Retinal Image Analysis: Concepts, Applications and Potential. Prog. Retin. Eye Res..

[4] Allen R.A., Straatsma B.R. (1961). Ocular Involvement in Leukemia and Allied Disorders. Arch. Ophthalmol..

[5] Kincaid M.C., Green W.R. (1983). Ocular and Orbital Involvement in Leukemia. Surv. Ophthalmol..

[6] Guyer D.R., Schachat A.P., Vitale S., Markowitz J.A., Braine H., Burke P.J., Karp J.E., Graham M. (1989). Leukemic Retinopathy. Ophthalmology.

[7] Karesh J.W., Goldman E.J., Reck K., Kelman S.E., Lee E.J., Schiffer C.A. (1989). A Prospective Ophthalmic Evaluation of Patients with Acute Myeloid Leukemia: Correlation of Ocular and Hematologic Findings. J. Clin. Oncol..

[8] Reddy S.C., Jackson N., Menon B.S. (2003). Ocular Involvement in Leukemia—A Study of 288 Cases. Ophthalmologica.

[9] Kashani A.H., Chen C.-L., Gahm J.K., Zheng F., Richter G.M., Rosenfeld P.J., Shi Y., Wang R.K. (2017). Optical Coherence Tomography Angiography: A Comprehensive Review of Current Methods and Clinical Applications. Prog. Retin. Eye Res..

[10] Spaide R.F., Fujimoto J.G., Waheed N.K., Sadda S.R., Staurenghi G. (2018). Optical Coherence Tomography Angiography. Prog. Retin. Eye Res..

[11] Potsaid B., Baumann B., Huang D., Barry S., Cable A.E., Schuman J.S., Duker J.S., Fujimoto J.G. (2010). Ultrahigh Speed 1050 nm Swept Source/Fourier Domain OCT Retinal and Anterior Segment Imaging at 100,000 to 400,000 Axial Scans per Second. Opt. Express.

[12] Choi W., Mohler K.J., Potsaid B., Lu C.D., Liu J.J., Jayaraman V., Cable A.E., Duker J.S., Huber R., Fujimoto J.G. (2013). Choriocapillaris and Choroidal Microvasculature Imaging with Ultrahigh Speed OCT Angiography. PLoS ONE.

[13] Kim A.Y., Chu Z., Shahidzadeh A., Wang R.K., Puliafito C.A., Kashani A.H. (2016). Quantifying Microvascular Density and Morphology in Diabetic Retinopathy Using Spectral-Domain Optical Coherence Tomography Angiography. Investig. Opthalmol. Vis. Sci..

[14] Zudaire E., Gambardella L., Kurcz C., Vermeren S. (2011). A Computational Tool for Quantitative Analysis of Vascular Networks. PLoS ONE.

[15] Sonoda S., Sakamoto T., Yamashita T., Shirasawa M., Uchino E., Terasaki H., Tomita M. (2014). Choroidal Structure in Normal Eyes and after Photodynamic Therapy Determined by Binarization of Optical Coherence Tomographic Images. Investig. Opthalmol. Vis. Sci..

[16] Agrawal R., Gupta P., Tan K.-A., Cheung C.M.G., Wong T.-Y., Cheng C.-Y. (2016). Choroidal Vascularity Index as a Measure of Vascular Status of the Choroid: Measurements in Healthy Eyes from a Population-Based Study. Sci. Rep..

[17] Lei J., Durbin M.K., Shi Y., Uji A., Balasubramanian S., Baghdasaryan E., Al-Sheikh M., Sadda S.R. (2017). Repeatability and Reproducibility of Superficial Macular Retinal Vessel Density Measurements Using Optical Coherence Tomography Angiography En Face Images. JAMA Ophthalmol..

[18] Scharf J., Freund K.B., Sadda S., Sarraf D. (2021). Paracentral Acute Middle Maculopathy and the Organization of the Retinal Capillary Plexuses. Prog. Retin. Eye Res..

[19] Yu D.-Y., Cringle S.J. (2001). Oxygen Distribution and Consumption within the Retina in Vascularised and Avascular Retinas and in Animal Models of Retinal Disease. Prog. Retin. Eye Res..

[20] Cicinelli M.V., Mastaglio S., Menean M., Marchese A., Miserocchi E., Modorati G., Bernardi M., Ciceri F., Bandello F. (2022). Retinal Microvascular Changes in Patients with Acute Leukemia. Retina.

[21] Yang L., Chen Y., Zhang Y., Shen T., Shen X. (2023). Changes in Retinal Circulation and Choroidal Thickness in Patients with Acute Myeloid Leukemia Detected by Optical Coherence Tomography Angiography. Front. Med..

[22] Zhou M., Hashimoto K., Wei D., Cai Y., Huang L., Shi X., Zhao M. (2024). Detection of Retinal Microvascular Changes with Optical Coherence Tomography Angiography in Patients with Acute Leukemia without Retinopathy. Ophthalmol. Ther..

[23] Lee J.H., Kim J.J., Hong S.Y., Kim G.-H., Kim J.-Y., Kim R.-Y., Kim M., Park Y.-G., Kim Y.-J., Cho B.-S. (2024). Analysis of Retinal and Choroidal Microvascular Changes Using Optical Coherence Tomography and Optical Coherence Tomography Angiography in Patients with Acute Leukemia. Graefes Arch. Clin. Exp. Ophthalmol..

[24] Xu S., Wang Y., Zhu P., Jiang S., Jiang Z. (2025). Observation of Choroidal and Retinal Changes in Acute Leukemia in Remission Using Ultra-Widefield Optical Coherence Tomography Angiography. Photodiagn. Photodyn. Ther..

[25] von Elm E., Altman D.G., Egger M., Pocock S.J., Gøtzsche P.C., Vandenbroucke J.P., Initiative S. (2007). The Strengthening the Reporting of Observational Studies in Epidemiology (STROBE) Statement: Guidelines for Reporting Observational Studies. Lancet.

[26] Khoury J.D., Solary E., Abla O., Akkari Y., Alaggio R., Apperley J.F., Bejar R., Berti E., Busque L., Chan J.K.C. (2022). The 5th Edition of the World Health Organization Classification of Haematolymphoid Tumours: Myeloid and Histiocytic/Dendritic Neoplasms. Leukemia.

[27] Arber D.A., Orazi A., Hasserjian R.P., Borowitz M.J., Calvo K.R., Kvasnicka H.-M., Wang S.A., Bagg A., Barbui T., Branford S. (2022). International Consensus Classification of Myeloid Neoplasms and Acute Leukemias: Integrating Morphologic, Clinical, and Genomic Data. Blood.

[28] Döhner H., Wei A.H., Appelbaum F.R., Craddock C., DiNardo C.D., Dombret H., Ebert B.L., Fenaux P., Godley L.A., Hasserjian R.P. (2022). Diagnosis and Management of AML in Adults: 2022 Recommendations from an International Expert Panel on Behalf of the ELN. Blood.

[29] Hoelzer D., Bassan R., Dombret H., Fielding A., Ribera J.M., Buske C., ESMO Guidelines Committee (2016). Acute Lymphoblastic Leukaemia in Adult Patients: ESMO Clinical Practice Guidelines for Diagnosis, Treatment and Follow-Up. Ann. Oncol..

[30] Gökbuget N., Boissel N., Chiaretti S., Dombret H., Doubek M., Fielding A., Foà R., Giebel S., Hoelzer D., Hunault M. (2024). Diagnosis, Prognostic Factors, and Assessment of ALL in Adults: 2024 ELN Recommendations from a European Expert Panel. Blood.

[31] Hong J., Ke M., Tan B., Lau A., Wong D., Yao X., Liu X., Schmetterer L., Chua J. (2020). Effect of Vessel Enhancement Filters on the Repeatability of Measurements Obtained from Widefield Swept-Source Optical Coherence Tomography Angiography. Sci. Rep..

[32] Koo T.K., Li M.Y. (2016). A Guideline of Selecting and Reporting Intraclass Correlation Coefficients for Reliability Research. J. Chiropr. Med..

[33] Shrout P.E., Fleiss J.L. (1979). Intraclass Correlations: Uses in Assessing Rater Reliability. Psychol. Bull..

[34] Al-Sheikh M., Tepelus T.C., Nazikyan T., Sadda S.R. (2017). Repeatability of Automated Vessel Density Measurements Using Optical Coherence Tomography Angiography. Br. J. Ophthalmol..

[35] Carpineto P., Mastropasqua R., Marchini G., Toto L., Di Nicola M., Di Antonio L. (2016). Reproducibility and Repeatability of Foveal Avascular Zone Measurements in Healthy Subjects by Optical Coherence Tomography Angiography. Br. J. Ophthalmol..

[36] Schindelin J., Arganda-Carreras I., Frise E., Kaynig V., Longair M., Pietzsch T., Preibisch S., Rueden C., Saalfeld S., Schmid B. (2012). Fiji: An Open-Source Platform for Biological-Image Analysis. Nat. Methods.

[37] Schneider C.A., Rasband W.S., Eliceiri K.W. (2012). NIH Image to ImageJ: 25 Years of Image Analysis. Nat. Methods.

[38] Arganda-Carreras I., Fernández-González R., Muñoz-Barrutia A., Ortiz-De-Solorzano C. (2010). 3D Reconstruction of Histological Sections: Application to Mammary Gland Tissue. Microsc. Res. Tech..

[39] Zahid S., Dolz-Marco R., Freund K.B., Balaratnasingam C., Dansingani K., Gilani F., Mehta N., Young E., Klifto M.R., Chae B. (2016). Fractal Dimensional Analysis of Optical Coherence Tomography Angiography in Eyes with Diabetic Retinopathy. Investig. Opthalmol. Vis. Sci..

[40] Benjamini Y., Hochberg Y. (1995). Controlling the False Discovery Rate: A Practical and Powerful Approach to Multiple Testing. J. R. Stat. Soc. Ser. B Stat. Methodol..

[41] Stone M.J., Bogen S.A. (2012). Evidence-Based Focused Review of Management of Hyperviscosity Syndrome. Blood.

[42] Stucki A., Rivier A.-S., Gikic M., Monai N., Schapira M., Spertini O. (2001). Endothelial Cell Activation by Myeloblasts: Molecular Mechanisms of Leukostasis and Leukemic Cell Dissemination. Blood.

[43] Luciano M., Krenn P.W., Horejs-Hoeck J. (2022). The Cytokine Network in Acute Myeloid Leukemia. Front. Immunol..

[44] Sanchez-Correa B., Bergua J.M., Campos C., Gayoso I., Arcos M.J., Bañas H., Morgado S., Casado J.G., Solana R., Tarazona R. (2013). Cytokine Profiles in Acute Myeloid Leukemia Patients at Diagnosis: Survival Is Inversely Correlated with IL-6 and Directly Correlated with IL-10 Levels. Cytokine.

[45] Aguayo A., Estey E., Kantarjian H., Mansouri T., Gidel C., Keating M., Giles F., Estrov Z., Barlogie B., Albitar M. (1999). Cellular Vascular Endothelial Growth Factor Is a Predictor of Outcome in Patients with Acute Myeloid Leukemia. Blood.

[46] Ley K., Laudanna C., Cybulsky M.I., Nourshargh S. (2007). Getting to the Site of Inflammation: The Leukocyte Adhesion Cascade Updated. Nat. Rev. Immunol..

[47] Incalza M.A., D’Oria R., Natalicchio A., Perrini S., Laviola L., Giorgino F. (2018). Oxidative Stress and Reactive Oxygen Species in Endothelial Dysfunction Associated with Cardiovascular and Metabolic Diseases. Vas. Pharmacol..

[48] Zamorano J.L., Lancellotti P., Rodriguez Muñoz D., Aboyans V., Asteggiano R., Galderisi M., Habib G., Lenihan D.J., Lip G.Y.H., Lyon A.R. (2016). 2016 ESC Position Paper on Cancer Treatments and Cardiovascular Toxicity Developed Under the Auspices of the ESC Committee for Practice Guidelines. Eur. Heart J..

[49] Chow A.Y., Chin C., Dahl G., Rosenthal D.N. (2006). Anthracyclines Cause Endothelial Injury in Pediatric Cancer Patients: A Pilot Study. J. Clin. Oncol..

[50] Koca S., Bozkurt E., Eroğul Ö., Yavaşoğlu F., Doğan M., Akdoğan M. (2022). Evaluation of Macular and Optic Disc Radial Peripapillary Vessel Density with Optical Coherence Tomography Angiography in Iron Deficiency Anemia. Photodiagn. Photodyn. Ther..

[51] Korkmaz M.F., Can M.E., Kazancı E.G. (2020). Effects of Iron Deficiency Anemia on Peripapillary and Macular Vessel Density Determined Using Optical Coherence Tomography Angiography on Children. Graefes Arch. Clin. Exp. Ophthalmol..

[52] Celik Dulger S., Cevik Kaya S., Fen T., Teke M.Y. (2024). Effects of Hemoglobin Concentration on Retinochoroidal Vascular Plexuses: An Optical Coherence Tomography Angiography Study. Int. Ophthalmol..

[53] Birkhoff W.A.J., van Manen L., Dijkstra J., De Kam M.L., van Meurs J.C., Cohen A.F. (2020). Retinal Oximetry and Fractal Analysis of Capillary Maps in Sickle Cell Disease Patients and Matched Healthy Volunteers. Graefes Arch. Clin. Exp. Ophthalmol..

[54] Clarke K., Mannath A., Anastasi M., Nasr M., Pan S., Balaskas K., Dinah C., Sarunic M.V., Asaria R. (2025). Optical Coherence Tomography Angiography as a Tool for Diagnosis and Monitoring of Sickle Cell Related Eye Disease: A Systematic Review and Meta-Analysis. Eye.

[55] FDA-NIH Biomarker Working Group (2016). BEST (Biomarkers, EndpointS, and Other Tools) Resource.

[56] Jodele S., Davies S.M., Lane A., Khoury J., Dandoy C., Goebel J., Myers K., Grimley M., Bleesing J., El-Bietar J. (2014). Diagnostic and Risk Criteria for HSCT-Associated Thrombotic Microangiopathy: A Study in Children and Young Adults. Blood.

[57] Coskuncan N.M., Jabs D.A., Dunn J.P., Haller J.A., Green W.R., Vogelsang G.B., Santos G.W. (1994). The Eye in Bone Marrow Transplantation. VI. Retinal Complications. Arch. Ophthalmol..

[58] Bossuyt P.M., Reitsma J.B., Bruns D.E., Gatsonis C.A., Glasziou P.P., Irwig L., Lijmer J.G., Moher D., Rennie D., de Vet H.C.W. (2015). STARD 2015: An Updated List of Essential Items for Reporting Diagnostic Accuracy Studies. BMJ.

[59] Collins G.S., Reitsma J.B., Altman D.G., Moons K.G.M. (2015). Transparent Reporting of a Multivariable Prediction Model for Individual Prognosis or Diagnosis (TRIPOD): The TRIPOD Statement. Ann. Intern. Med..

[60] Wolff R.F., Moons K.G.M., Riley R.D., Whiting P.F., Westwood M., Collins G.S., Reitsma J.B., Kleijnen J., Mallett S., for the PROBAST Group (2019). PROBAST: A Tool to Assess the Risk of Bias and Applicability of Prediction Model Studies. Ann. Intern. Med..

[61] Alam M., Thapa D., Lim J.I., Cao D., Yao X. (2017). Computer-Aided Classification of Sickle Cell Retinopathy Using Quantitative Features in Optical Coherence Tomography Angiography. Biomed. Opt. Express.

[62] London A., Benhar I., Schwartz M. (2013). The Retina as a Window to the Brain—From Eye Research to CNS Disorders. Nat. Rev. Neurol..

[63] Gulshan V., Peng L., Coram M., Stumpe M.C., Wu D., Narayanaswamy A., Venugopalan S., Widner K., Madams T., Cuadros J. (2016). Development and Validation of a Deep Learning Algorithm for Detection of Diabetic Retinopathy in Retinal Fundus Photographs. JAMA.

