# Supplementary material for: Retinal Microvascular Changes Associated with Concurrent Morphologic/Hematologic Response to Induction Therapy in Acute Leukemia: An Exploratory Prospective Wide-Field Swept-Source OCT Angiography Study
Source: Diagnostics (Basel). 2026 Sep 11;16(18):2940. doi: 10.3390/diagnostics16182940 (PMC13605835; doi:10.3390/diagnostics16182940)
Supplement: Supplementary file 1 [file diagnostics-16-02940-s001.zip › diagnostics-4542796-supplementary.pdf]

Supplementary Materials

Retinal Microvascular Changes Associated with Concurrent Morphologic/Hematologic Response to Induction Therapy in Acute Leukemia: An Exploratory Prospective Wide-Field Swept-Source OCT Angiography Study

Supplementary Methods

S1. Wide-Field SS-OCTA Acquisition Standard Operating Procedure

All scans were performed on a single 400-kHz swept-source OCTA device (BM-400K BMizar; TowardPi Medical Technology, Beijing, China) with a fixed software version throughout the study (version/build registered at acquisition start, held fixed throughout, and archived with the imaging acquisition log). Additional acquisition parameters (B-scan count and spacing, acquisition time, and axial resolution) were likewise registered at acquisition start and archived with the imaging acquisition log; the 16 × 16 mm protocol comprised 1024 B-scan positions × 1024 A-scans each (1948 depth pixels; nominal acquisition time ≈2.6 s at 400 kHz; nominal lateral sampling 15.625 μm/pixel), and up to two repeat scans per eye per time point were permitted. The 16 × 16 mm scan pattern was centered on the fovea and covered the optic disc, allowing simultaneous macular and peripapillary assessment without montage. All OCTA examinations were performed before pupillary dilation through natural pupils, by technicians trained under a unified standard operating procedure. Each eye was scanned independently and saved under a unique identifier; T0 and T1 scans of the same patient used the identical device and protocol. The 16 × 16 mm field was chosen over larger (e.g., 24 × 20 mm) protocols because, at fixed A-scan density, wider fields reduce peripheral sampling density and signal-to-noise ratio, compromising the quantitative repeatability required for longitudinal comparison. The device operates at a center wavelength of 1050 nm; manufacturer, model, software version, and optical specifications were registered at acquisition start, held fixed throughout the study, and are archived with the study database. Automated layer segmentation used manufacturer-defined slab boundaries (superficial capillary plexus [SCP]: internal limiting membrane to the inner plexiform/inner nuclear layer [IPL/INL] boundary; deep capillary plexus [DCP]: IPL/INL boundary to the outer plexiform layer [OPL]). The device's projection-resolved artifact-removal algorithm and motion correction were enabled for all scans. Optic-disc, large-vessel, and low-signal/invalid-area masks were applied uniformly, and vessel density was computed as the perfused-pixel area divided by the valid analyzed area. Output angiograms were 1024 × 1024 pixels over 16 × 16 mm (nominal 15.625 μm/pixel). T0 and T1 scans of the same eye used the identical device, protocol, and fovea-centered acquisition; no automated follow-up image registration was applied.

S2. Image Quality Control

Image quality was managed with the device output IQ score (integer 1–10); IQ ≥ 7 was required for inclusion. Each eye at each time point was tracked in a four-state system: PASS (IQ ≥ 7), REPEAT (rescan pending), EXCLUDE (persistently

substandard after rescans), and MISSING. On-site rescan triggers at acquisition were IQ < 7, significant motion artifacts (motion artifact score > 2), defocus, or image truncation; up to two repeat scans were allowed per eye per time point. All state changes were logged in the study database. Automated segmentation was independently reviewed by two trained readers; correctable segmentation errors were manually adjusted with re-computation of metrics, and uncorrectable errors led to exclusion of the corresponding metric for that eye. Reader training targets were an IQ-score agreement rate ≥85%, FAZ-abnormality recognition accuracy ≥90%, and a parallel-reading ICC ≥0.80 (operational training targets fixed at reader onboarding). Between-eye consistency targets were ICC 0.75–0.95 for vessel density metrics, 0.70–0.90 for FAZ metrics, and ≥0.80 for SFCT. Achieved between-eye concordance is reported in the main text (Section 3.6) and Table S5; formal inter-reader and test-retest repeatability studies were not performed and are acknowledged as limitations. At the final offline eligibility review, artifact thresholds for exclusion or correction—applied as operational rules during acquisition and formally consolidated in the current SOP thereafter—were: motion artifacts involving > 5% of the analyzed area, uncorrectable large-vessel projection involving > 10%, segmentation boundary shift > 20 μm or involving the central 3 mm, and invalid areas > 10%. The on-site rescan triggers and the offline thresholds were applied sequentially—the offline review being decisive—and all corrections were logged in the study database. Among 302 baseline eye scans, 283 (93.7%) passed quality control (19 excluded after rescans); all 154 T1 scans passed. Among analyzed eyes, IQ scores were: T0 mean 8.90 ± 1.09, median 9 (IQR 8–10; group means: AML 8.65, ALL 8.88, HC 9.25); T1 mean 8.45 ± 1.08, median 8 (IQR 8–9; group means: AML 8.36, ALL 8.58). Patient-level ΔIQ among completers averaged −0.24 ± 1.51 (CR/CRi −0.15 vs PR/NR −0.46). The current SOP version was formally consolidated after completion of data collection, operationalizing the rules applied during the study; the device software version and acquisition parameters were registered at acquisition start and are archived with the imaging acquisition log.

S3. Fiji/ImageJ Deep Capillary Plexus Morphology Pipeline

DCP en face images (16 × 16 mm, 1024 × 1024 pixels, 8-bit; 1 pixel = 15.625 μm) were analyzed with Fiji (version 2.18.0; ImageJ core 1.54p) using a fixed six-step pipeline: (1) scale calibration (1024 pixels = 16 mm); (2) background subtraction (rolling ball radius 50 pixels) and despeckling; (3) Otsu auto-threshold binarization (Convert to Mask; second despeckle); (4) manual optic-disc masking (elliptical selection archived in ROI Manager; disc area subtracted from the 256 mm^2 total so that analyzed area = 256 minus disc area); (5) skeletonization (Skeletonize 2D/3D) and topology extraction with AnalyzeSkeleton (prune cycle method = none; prune ends off; detailed info on), yielding vessel length density (VLD = total skeleton branch length/analyzed area, mm/mm^2) and junction density (total junctions/analyzed area, n/mm^2); (6) box-counting fractal dimension (Fractal Box Count; black background; box sizes 2–128 pixels; double-log linear fit with $R^2 > 0.99$ required). All parameters remained unchanged throughout the study. Internal process targets for the agreement of repeat measurements, expressed as absolute

differences between repeats, were |ΔVLD| < 1.0 mm/mm^2, |ΔFD| < 0.08, and |Δjunction density| < 1.5 n/mm^2. These were process targets used during pipeline development; no formal repeat-measurement substudy was performed, and these values are not empirical test-retest results. These morphology metrics are relative image-processing-level quantifiers intended for between-group and within-subject comparisons, not absolute anatomic equivalents. They were exploratory image-processing-derived parameters and were not intended as clinical biomarkers. No individual axial-length magnification correction was applied; the nominal device calibration (15.625 μm/pixel) was used for all eyes, and eyes with axial length > 26.00 mm were excluded to limit scale error.

S4. Choroidal Quantification

Structural OCT provided SFCT and central macular thickness. Choroidal density metrics (choriocapillaris vessel density [VDCC]; medium/large choroidal vessel density [VDMLCV]) were obtained from device angiographic outputs. Volumetric choroidal indices — three-dimensional choroidal vascularity index (3D-CVI, choroidal vessel volume/total choroidal volume), choroidal vessel volume per unit area (CVV/a), and choroidal stromal volume per unit area (CSV/a) — were generated by the device's built-in software from automated choroidal segmentation and quantification, a device implementation of the choroidal binarization and vascularity-index methodology of Sonoda et al. and Agrawal et al. [15,16], computed as: 3D-CVI = choroidal vessel volume / total choroidal volume; CVV/a = choroidal vessel volume normalized to the analyzed area; and CSV/a = choroidal stromal volume per unit area. Choroidal boundaries were the outer border of the retinal pigment epithelium–Bruch's membrane complex (inner) and the choroidoscleral interface (outer); automated segmentation was visually verified, and correctable errors were manually adjusted with re-computation before volume computation.

Supplementary Tables

Table S1. Patient-level prevalence of fundus manifestations at diagnosis (T0).

Fundus manifestation	AML (n = 58), n (%)	ALL (n = 53), n (%)	P value
Retinal hemorrhage	13 (22.4)	9 (17.0)	0.632
Roth spots	5 (8.6)	4 (7.5)	1.000
Cotton-wool spots	8 (13.8)	5 (9.4)	0.562
Retinal vascular abnormalities	5 (8.6)	4 (7.5)	1.000
Disc edema	2 (3.4)	1 (1.9)	1.000
Macular involvement	4 (6.9)	2 (3.8)	0.681

Fundus manifestation	AML (n = 58), n (%)	ALL (n = 53), n (%)	P value
Leukemic infiltration	2 (3.4)	1 (1.9)	1.000

Chi-square or Fisher exact test; p values are reported to three decimal places (values below 0.01 are denoted as P < 0.01); no asterisk annotation is used. Overall retinal hemorrhage prevalence 19.8% (22 patients/39 eyes); grade 1/2/3 distribution among the 39 affected eyes was 17/39 (43.6%), 18/39 (46.2%), and 4/39 (10.3%). Hemorrhage grade (ordinal 0–3; no hemorrhage scored 0; patient level = maximum across eyes) correlated negatively with baseline Hb (Spearman $r_s = -0.316$, $P < 0.01$, n = 111) and with DCP-VD (patient level $r_s = -0.280$, $P < 0.01$; eye level across the 203 quality-eligible eyes $r_s = -0.236$, $P < 0.01$).

Table S2. Full foveal avascular zone metrics at T0.

Variable	AML (n = 58)	ALL (n = 53)	HC (n = 40)	P value
SCP-FAZ area, mm^2	0.36 (0.31, 0.41)	0.37 ± 0.07	0.35 ± 0.04	0.320
DCP-FAZ area, mm^2	0.46 (0.40, 0.53)	0.46 ± 0.08	0.41 ± 0.04	< 0.01
Whole-FAZ area, mm^2	0.34 ± 0.05	0.34 ± 0.03	0.33 ± 0.03	0.133
SCP-FAZ perimeter, mm	3.02 ± 0.30	2.96 ± 0.27	2.88 (2.74, 3.06)	0.128
DCP-FAZ perimeter, mm	3.55 ± 0.32	3.50 ± 0.27	3.41 ± 0.20	0.070
Whole-FAZ perimeter, mm	2.66 (2.55, 2.79)	2.62 ± 0.16	2.60 ± 0.15	0.201

Kruskal-Wallis or one-way ANOVA with Bonferroni-adjusted pairwise comparisons, selected by Shapiro-Wilk normality assessment; DCP-FAZ area differed across groups (P < 0.01; AML vs HC P < 0.01; ALL vs HC P < 0.01) and remained significant after Benjamini-Hochberg correction within the 20-comparison baseline family (q < 0.01), whereas the other five FAZ metrics did not (q range 0.116–0.337). Effect sizes: whole-FAZ area $\eta^2 = 0.027$ (ANOVA); DCP-FAZ area $\varepsilon^2 = 0.110$ (rank-based). Magnitudes across the two effect-size definitions are not directly comparable; P values are reported to three decimal places (values below 0.01 are denoted as P < 0.01); no asterisk annotation is used.

Table S3. Full choroidal and structural OCT metrics at T0.

Variable	AML (n = 58)	ALL (n = 53)	HC (n = 40)	P value

Variable	AML (n = 58)	ALL (n = 53)	HC (n = 40)	P value
VDCC, %	46.75 (40.62, 51.38)	46.50 (44.00, 52.50)	50.14 ± 4.63	< 0.01
VDMLCV, %	53.25 (50.00, 60.75)	56.00 (50.00, 63.00)	58.20 ± 7.50	0.178
3D-CVI, %	33.50 (32.00, 34.00)	32.00 (29.00, 37.00)	34.99 ± 2.91	< 0.01
CVV/a, µm	75.00 (65.25, 80.50)	77.00 (62.50, 83.00)	79.50 (72.50, 87.50)	0.013
CSV/a, µm	156.00 (141.12, 171.50)	156.50 (128.50, 168.50)	151.00 (132.38, 160.00)	0.088
SFCT, µm	256.00 (224.25, 268.88)	263.84 ± 42.74	261.50 (238.38, 287.88)	0.019
CMT, µm	244.75 (238.00, 255.25)	244.92 ± 11.50	247.00 (241.50, 252.75)	0.384

Kruskal-Wallis test with Bonferroni-adjusted pairwise comparisons; VDCC, 3D-CVI (both P < 0.01), and CVV/a (P = 0.013) differed across groups and remained significant after Benjamini-Hochberg correction within the 20-comparison baseline family (q = 0.018, q < 0.01, and q = 0.031, respectively), as did SFCT (P = 0.019, q = 0.038; AML lower than ALL, Bonferroni P = 0.037); VDMLCV (P = 0.178), CSV/a (P = 0.088), and central macular thickness (P = 0.384) did not differ. SFCT correlated positively with baseline Hb (patients, n = 111: Spearman r_s = +0.217, P = 0.022) and with CVV/a (all participants, n = 151: r_s = +0.723, P < 0.01), and negatively with age (patients, n = 111: r_s = −0.318, P < 0.01) and axial length (all participants, n = 151: r_s = −0.270, P < 0.01); P values are reported to three decimal places (values below 0.01 are denoted as P < 0.01); no asterisk annotation is used.

Table S4. Full DCP morphology metrics at T0 (Fiji pipeline).

Variable	AML (n = 58)	ALL (n = 53)	HC (n = 40)	P value
DCP-VLD, mm/mm^2	8.56 (6.91, 9.90)	8.38 ± 1.75	9.92 ± 1.29	< 0.01
DCP-FD	1.54 (1.37, 1.68)	1.55 (1.42, 1.70)	1.69 ± 0.05	< 0.01
DCP junction density, n/mm^2	35.84 ± 9.17	35.92 (30.05, 40.30)	38.66 (32.30, 46.67)	0.044

Kruskal-Wallis test with Bonferroni-adjusted pairwise comparisons; DCP-VLD and DCP-FD differed across groups (both P < 0.01; AML vs HC and ALL vs HC both P < 0.01) and survived Benjamini-Hochberg correction within the 20-comparison baseline family (both q < 0.01); DCP junction density differed nominally (P = 0.044; ALL vs HC P = 0.035) but did not survive correction (q = 0.080). Effect sizes (ε^2, descriptive, rank-based): DCP-VLD 0.142, DCP-FD 0.135, junction density 0.029. Junction density, VLD, and FD did not differ between AML and ALL after Bonferroni adjustment (all P = 1.000); P values are reported to three decimal places (values below 0.01 are denoted as P < 0.01); no asterisk annotation is used.

Table S5. Sensitivity and supplementary analyses summary (same-eye pairing; quality-controlled eyes).

Analysis	Model / statistic	Key estimate (95% CI)	P value
AML-vs-ALL confounding adjustment	OLS (n = 151: 58 AML, 53 ALL, 40 HC); formula: DCP-VD ~ group (3-level; ref: ALL) + age + sex + Hb + image quality score; only quality-eligible eyes averaged	B = −0.801 (−1.945 to +0.344)	0.169
Same, eye-level	Linear model, 203 eligible patient eyes; formula: DCP-VD ~ group (ref: ALL) + age + sex + Hb; cluster-robust sandwich SEs by patient (CR0; normal-approximation CIs)	B = −0.699 (−1.852 to +0.455)	0.235
Change-baseline coupling	ANCOVA (n = 77); formula: T1 DCP-VD ~ response group (exposure; ref: PR/NR) + T0 DCP-VD + ΔHb + age + sex; R^2 = 0.950	remission B = +0.56 (0.16–0.97); ΔHb B = +0.069 (0.036–0.103)	< 0.01 / < 0.01

Analysis	Model / statistic	Key estimate (95% CI)	P value
Eye-level longitudinal	Linear model, 143 eligible T1 eye scans (77 patients; same-eye rule); formula: ΔDCP-VD ~ response group (ref: PR/NR); cluster-robust sandwich SEs by patient (CR0; normal-approximation CIs)	B = +1.02 (0.65 to 1.39)	< 0.01
T1 window	Days-from-T0-to-T1 vs ΔDCP-VD	r_s = +0.251	0.028
T1 window, adjusted	OLS: ΔDCP-VD ~ days (T0 to T1) + response group (ref: PR/NR)	days B = +0.019/day; remission B = +0.935	0.103; < 0.01
Hemorrhage grade vs DCP-VD	Spearman (patient level n = 111 / eye level n = 203 eligible eyes)	r_s = −0.280 / r_s = −0.236	< 0.01 / < 0.01; patient-level cluster bootstrap (2000 resamples): 91.7% of resamples P < 0.05
Hemorrhage grade vs baseline Hb	Spearman (n = 111)	r_s = −0.316	< 0.01

Analysis	Model / statistic	Key estimate (95% CI)	P value
Between-eye consistency (T0)	ICC(2,1); pooled 132 both-eligible pairs; patients only 92 pairs	pooled: IRVD 0.847, DCP-VD 0.607; patients only: IRVD 0.819 (95% CI 0.743–0.879), DCP-VD 0.585 (0.435–0.706); pooled values exceed patient-only values because of between-group heterogeneity and are not interpreted as test-retest or inter-reader reliability	-
Four-category response breakdown of ΔDCP-VD	mean ± SD / median (IQR), patient level	CR 1.66 ± 0.72 (n = 25); CRi 1.00 (1.00, 1.50) (n = 29); PR 0.41 ± 0.71 (n = 16); NR 0.71 ± 1.07 (n = 7)	Bonferroni-adjusted MWU: CR vs CRi 0.240; PR vs NR 1.000
Baseline-severity-adjusted response comparison	OLS (n = 77): ΔDCP-VD ~ response group + baseline Hb + baseline PLT + platelet transfusion; R^2 = 0.307	remission B = +0.79 (0.37 to 1.21)	< 0.01

Analysis	Model / statistic	Key estimate (95% CI)	P value
Image quality by time point	device IQ score (1–10), analyzed eyes	T0 mean 8.90 ± 1.09 (median 9, IQR 8–10); T1 mean 8.45 ± 1.08 (median 8, IQR 8–9); patient-level ΔIQ among completers −0.24 ± 1.51 (CR/CRi 0.00 (−1.38, 1.00), PR/NR −0.46 ± 1.54)	-
T1-to-marrow interval	days from T1 scan to response-assessment bone marrow	median 2 (IQR 1–4); range −3 to 7; 100% within ±7 days	-
Response-category distribution by subtype	n by category	AML: CR 15, CRi 16, PR 10, NR 3; ALL: CR 10, CRi 13, PR 6, NR 4	-
Subtype difference in ΔDCP-VD (longitudinal set)	t test; AML n = 44, ALL n = 33	+1.27 ± 1.01 vs +1.06 ± 0.76 percentage points; within CR/CRi: +1.58 vs +1.33; within PR/NR: +0.54 vs +0.45	0.317; 0.246; 0.806
ΔIQ-adjusted response comparison	OLS (n = 77): ΔDCP-VD ~ response group + paired-eye ΔIQ; R^2 = 0.244	remission B = +0.96 (0.56 to 1.36); ΔIQ B = +0.04, P = 0.554	< 0.01
Excluding eyes with baseline retinal hemorrhage	patient-level same-eye analysis (n = 60: CR/CRi 42, PR/NR 18)	between-group difference 0.96 percentage points (+1.43 vs +0.47)	< 0.01

Analysis	Model / statistic	Key estimate (95% CI)	P value
Attrition balance, extended panel	completers (n = 77) vs lost to follow-up (n = 34); standardized mean differences	age −0.17; Hb −0.31; WBC +0.08; PLT −0.01; marrow blasts −0.27; baseline DCP-VD −0.15; AML proportion +0.32; male proportion −0.03	P range 0.129–1.000
Eye-level longitudinal (mixed model)	Linear mixed-effects model, 286 eye-time observations (143 same-eye pairs × 2 time points); patient random intercept, maximum-likelihood estimation; time × response interaction	interaction B = +1.02 (0.20–1.84)	0.015
Equal-variance assumption sensitivity	Welch-corrected two-sample t test (main comparison)	between-group difference 0.97 percentage points (0.56–1.38); t = 4.787	< 0.01

Analysis	Model / statistic	Key estimate (95% CI)	P value
Attrition sensitivity: inverse probability weighting	111-patient logistic model for T1 completion (baseline age, sex, subtype, Hb, WBC, PLT, marrow blasts, DCP-VD); C statistic 0.665; stabilized weights truncated at 1st/99th percentiles (0.76–1.63); effective sample size 73.9; robust standard errors	weighted between-group difference +1.04 percentage points (0.66–1.42)	< 0.01
Attrition sensitivity: multiple imputation	MICE, m = 20; chained equations jointly imputing response status (logistic) and ΔDCP-VD (linear) under MAR; Rubin's rules	pooled between-group difference +0.96 percentage points (0.55–1.37)	95% CI excludes 0
Attrition sensitivity: δ-tipping	non-completer ΔDCP-VD shifted by δ (0 to −15 percentage points, step 0.25) relative to same-subtype completers; 500 Monte Carlo replications per grid point	significance lost only at δ ≤ −1.75 percentage points (δ* = −1.75)	P ≥ 0.05 only for δ ≤ −1.75

P values are reported to three decimal places (values below 0.01 are denoted as P < 0.01); no asterisk annotation is used. q values are Benjamini-Hochberg FDR estimates as specified in the main text.

Table S6. Exploratory ROC analysis details: ΔDCP-VD discriminating remission (CR/CRi) from nonremission (PR/NR) (n = 77; CR/CRi 54, PR/NR 23; same-eye pairing).

Characteristic	Estimate
Apparent in-sample AUC (bootstrap 95% CI, 5000 resamples; not corrected for selection optimism)	0.799 (0.674–0.907)
Post hoc sample-specific Youden point (ΔDCP-VD; descriptive only — not a clinical cutoff)	+1.00 percentage points
Youden index	0.541
Sensitivity	88.9% (48/54); 95% CI 77.4–95.8
Specificity	65.2% (15/23); 95% CI 42.7–83.6
Positive predictive value	85.7% (48/56); 95% CI 73.8–93.6
Negative predictive value	71.4% (15/21); 95% CI 47.8–88.7
Positive likelihood ratio (LR+)	2.56 (95% CI 1.45–4.51)
Negative likelihood ratio (LR−)	0.170 (95% CI 0.076–0.383)
Sample-specific descriptive operating rule	ΔDCP-VD ≥ +1.00 percentage points
2 × 2 table (at the sample-specific point ≥ +1.00)	TP 48, FN 6, TN 15, FP 8

Estimates are derived in-sample from the same cohort used to define the response groups; they represent exploratory discrimination performance and are hypothesis-generating, not diagnostic accuracy. Because imaging and response were assessed concurrently at T1, the analysis reflects association, not prediction. External validation in an independent cohort is required before any threshold-based interpretation. Confidence intervals for proportions are two-sided Clopper–Pearson exact 95% intervals; likelihood-ratio intervals are log-method approximations.

Table S7. Treatment exposures during the T0–T1 interval by subtype.

Variable	AML (n = 58)	ALL (n = 53)	P value
RBC transfusion, U	4.52 ± 2.38	4.42 ± 2.58	0.828
Platelet transfusion, doses	3.00 (2.00, 4.00)	3.75 ± 1.72	0.238
First-course anthracycline, mg/m^2	180.00 (135.00, 180.00)	90.00 (60.00, 120.00)	< 0.01

Treatment exposures are T0–T1 process variables and are reported separately from baseline characteristics (Table 1); response-group distributions are shown in Table 2. Data are mean ± SD or median (interquartile range); comparisons by

independent-samples t test or Mann-Whitney U test; P values are reported to three decimal places (values below 0.01 are denoted as $P < 0.01$); no asterisk annotation is used. T1 denotes the clinical first-response-assessment time point (anchored to the response-assessment marrow examination), not the T1 imaging scan; 77 of the 111 patients (44 AML, 33 ALL) completed T1 imaging.

Supplementary Figure

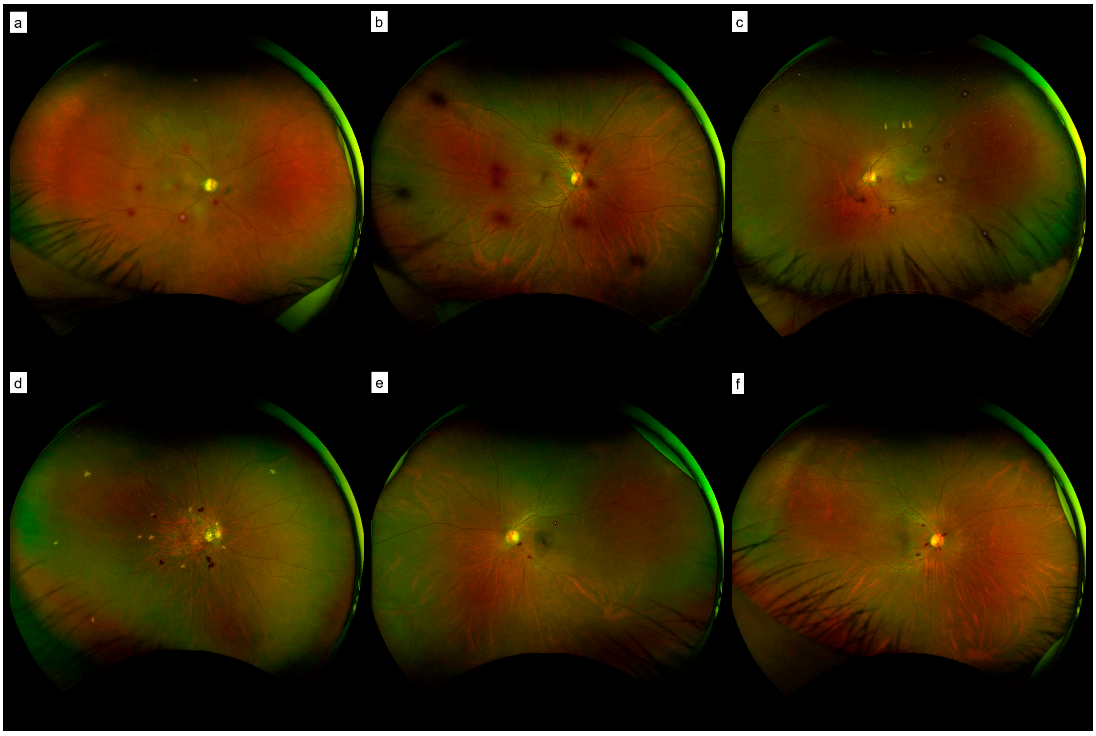

Figure S1. Representative fundus manifestations at diagnosis (T0), shown by ultra-widefield color fundus photography. (a) Mild retinal hemorrhage; (b) moderate patchy retinal hemorrhage; (c) Roth spots (white-centered hemorrhages); (d) cotton-wool spots; (e) suspected macular involvement (no structural OCT confirmation available; not scored as confirmed macular edema); (f) mild disc edema. Manifestations were predominantly mild and scattered; severe findings (disc edema, macular involvement, leukemic infiltration) were rare.

Supplementary Statistical Note on the Exploratory Discrimination Analysis

The exploratory receiver operating characteristic (ROC) analysis evaluated ΔDCP-VD as a continuous marker discriminating remission (CR/CRi, $n = 54$) from nonremission (PR/NR, $n = 23$), using quality-controlled eyes with same-eye pairing. The area under the curve (AUC) was computed by the rank-based (Mann-Whitney) method; the 95% confidence interval was estimated with 5000 non-stratified bootstrap resamples (percentile method; Python 3.13, NumPy random generator, seed 20260726). The AUC is an apparent, patient-level, in-sample estimate (0.799, 95% CI 0.674–0.907). The post hoc sample-specific operating point was derived

with the Youden index within the same cohort; its sensitivity, specificity, and predictive values were likewise derived and evaluated in this cohort; it was not internally or externally validated and should not be interpreted as a usable clinical threshold. The bootstrap confidence interval quantifies sampling variability of the apparent AUC only and does not correct optimism arising from post hoc marker selection, operating-point derivation, and performance estimation on the same data. Positive and negative predictive values depend on the 54/77 remission proportion of this sample and are not transportable. These supplementary analyses quantify the strength of the concurrent association but do not alter the exploratory, hypothesis-generating nature of the discrimination analysis, and external validation in an independent cohort is required.
